# The macrophage-intrinsic MDA5/IRF5 axis drives HIV-1 intron-containing RNA-induced inflammatory responses

**DOI:** 10.1172/JCI187663

**Published:** 2025-06-10

**Authors:** Sita Ramaswamy, Hisashi Akiyama, Jacob Berrigan, Andrés A. Quiñones-Molina, Alex J. Olson, Yunhan Chen, YanMei Liang, Andrew J. Henderson, Archana Asundi, Manish Sagar, Suryaram Gummuluru

**Affiliations:** 1 Department of Virology, Immunology & Microbiology, Boston University Chobanian & Avedisian School of Medicine, Boston, Massachusetts, USA.; 2 Section of Infectious Diseases, Department of Medicine, Boston Medical Center, Boston, Massachusetts, USA.

**Keywords:** AIDS/HIV, Aging, Inflammation, Innate immunity, Macrophages

## Abstract

Despite effective antiretroviral therapy, transcriptionally competent HIV-1 reservoirs remain and contribute to persistent immune activation in people living with HIV (PWH). HIV-1–infected macrophages are important mediators of chronic innate immune activation, though the mechanisms remain unclear. We previously reported that nuclear export and cytoplasmic expression of HIV-1 intron-containing RNA (icRNA) activates mitochondrial antiviral signaling (MAVS) protein–mediated type I IFN responses in macrophages. In this study, we demonstrate an essential role of melanoma differentiation–associated protein 5 (MDA5) in sensing HIV-1 icRNA and promoting MAVS-dependent interferon regulatory factor 5 (IRF5) activation in macrophages. Suppression of MDA5 but not retinoic acid–inducible gene I expression nor disruption of the endosomal TLR pathway abrogated HIV-1 icRNA-induced type I IFN responses and IP-10 expression in macrophages. Furthermore, induction of IP-10 in macrophages upon HIV-1 icRNA sensing by MDA5 was dependent on IRF5. Additionally, monocytes and monocyte-derived macrophages (MDMs) from older (>50 years) individuals exhibited constitutively higher levels of IRF5 expression compared with younger (<35 years) individuals, and HIV-1 icRNA-induced IP-10 expression was significantly enhanced in older macrophages, which was attenuated upon ablation of IRF5 expression, suggesting that IRF5 functions as a major mediator of proinflammatory response downstream of MDA5-dependent HIV-1 icRNA sensing, dysregulation of which might contribute to chronic inflammation in older PWH.

## Abstract 2

In HIV-infected individuals, innate immune activation and inflammation persist despite anti-retroviral therapy, with many immunological defects similar to ones observed in the aged population. In this manuscript, we provide evidence that advanced age and HIV-1 RNA expression-induced inflammatory signaling pathway in macrophages synergistically promote type I IFN responses and inflammation.

## Graphical abstract

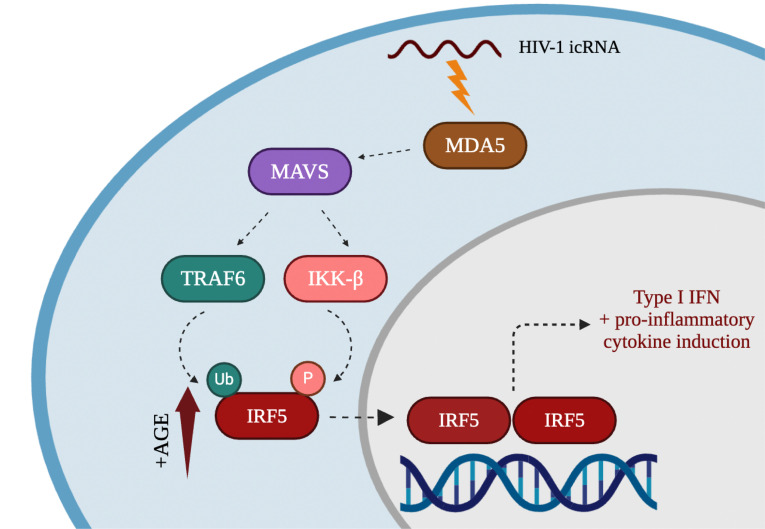

## Introduction

HIV-1 remains a global burden with approximately 39 million people living with HIV (PWH) as of 2022 (UNAIDS/WHO, 2023). Although antiretroviral therapy (ART) has been successful in suppressing virus replication to undetectable levels and extending the lifespan of PWH, systemic inflammatory responses in ART-suppressed PWH remain elevated (1–3). Additionally, chronic systemic inflammation increases the risk for diseases associated with aging, such as neurocognitive disorders, cancer, and coronary artery disease (4–7), which account for most of the morbidity and mortality in virologically suppressed PWH (8) and 5 to 10 year loss in life expectancy compared with risk-adjusted people without HIV (9).

The exact mechanism by which chronic HIV-1 infection contributes to systemic inflammation in virologically suppressed PWH is unclear. It is well established that early in the course of infection, viral reservoirs are established in long-lived cell populations, such as memory CD4^+^ T cells and macrophages (10, 11). Tissue-resident macrophages located in peripheral lymphoid tissues, liver, brain, lungs, and mucosal tissues harbor HIV RNA and DNA and remain persistently infected even during suppressive ART (12). While ART reduces peripheral viremia, currently available ART regimens do not suppress viral transcription, and a subset of cells containing integrated provirus remain transcriptionally competent (13). As a result, both viral RNAs and proteins have also been detected in lymph nodes and CNS of ART-suppressed patients (14) and are hypothesized to act as pathogen-associated molecular patterns (PAMPs) that can induce persistent inflammatory responses. In concordance with this hypothesis, several recent studies have utilized patient cohort samples to show correlations of HIV-1 RNA/DNA persistence with elevated innate immune markers and chronic inflammation (15–17).

Chronic immune activation as a consequence of persistent HIV-1 infection is compounded by the low-grade chronic inflammation that occurs during aging, and a combination of these phenotypes is referred to as HIV “inflammaging” (18, 19). This innate immune aging phenotype is proposed to be mediated by cells in the tissue and stroma responding to diverse stimuli within the tissue microenvironment and at the systemic level (20, 21). Tissue-resident macrophages are potentially the primary sensors of cellular injury or infection and are responsible for elevated secretion of infection- or injury-induced inflammatory cytokines, such as IL-6, sCD14, and sCD163 (22). In addition, aged macrophages have diminished phagocytic capabilities, resulting in a failure to resolve inflammation (23, 24), and have been primarily associated with tissue pathology and age-associated end-organ diseases (25, 26). Several studies have indicated that the population of monocytes and macrophages increases with age in many tissue compartments, and the polarization of these cells toward an inflammatory phenotype is also enhanced in older people without HIV (27) and in older PWH (28). How HIV infection contributes to premature and accelerated aging of the innate immune system has remained unclear.

Previous findings from our group and others showed that cytoplasmic expression of HIV-1 intron-containing RNA (icRNA) triggers induction of interferon-stimulated gene (ISG) expression and type I IFNs in macrophages and dendritic cells (29, 30). Nuclear export of HIV-1 icRNA is dependent on the HIV-1 protein Rev, which recognizes and binds a structured domain of HIV-1 RNA referred to as the Rev Response Element (RRE) (31). Upon Rev binding to the RRE, a nuclear export factor, CRM1, is recruited to Rev and the Rev/RRE complex is shuttled out of the nucleus into the cytosol (32). Sensing of HIV-1 icRNA in the cytoplasm results in the activation of the mitochondrial antiviral signaling (MAVS) protein, initiating a downstream signaling response and innate immune activation (29, 30).

Retinoic acid–inducible gene I (RIG-I)–like receptors (RLRs) are cytosolic sensors that detect dsRNAs and mediate an IFN response to viral infections (33). RLRs include RIG-I, melanoma differentiation–associated protein 5 (MDA5), and laboratory of genetics and physiology 2, though only RIG-I and MDA5 have been shown to sense dsRNAs in a sequence-independent manner (34). Recent work has implicated MDA5 as a sensor of unspliced HIV-1 RNA (35). Oligomerization of MAVS allows for the recruitment of various E3 ubiquitin ligases, TNF receptor–associated factors (TRAFs), and cytosolic kinases, which in turn leads to activation of transcription factors, such as NF-κB and interferon regulatory factors (IRFs) (36). In this study, we confirm the critical role of MDA5 as a sensor of HIV-1 icRNA in human macrophages and IRF5 in mediating MDA5/MAVS-dependent type I IFN responses and proinflammatory cytokine production. Interestingly, monocytes and MDMs from older donors (>50 years old [yo]) displayed elevated levels of constitutive IRF5 expression compared with younger donors (<35 yo), which correlated with higher levels of HIV-1 icRNA-induced interferon γ inducible protein-10 (IP-10) production in MDMs. Taken together, these findings offer mechanistic insights into the understanding of how persistently expressed HIV-1 icRNA can promote chronic expression of proinflammatory cytokines and might contribute to inflammaging in older PWH.

## Results

### Rev/CRM1-dependent nuclear export and MAVS are required for HIV-1 icRNA sensing in THP-1/PMA macrophages.

**Figure 1:**
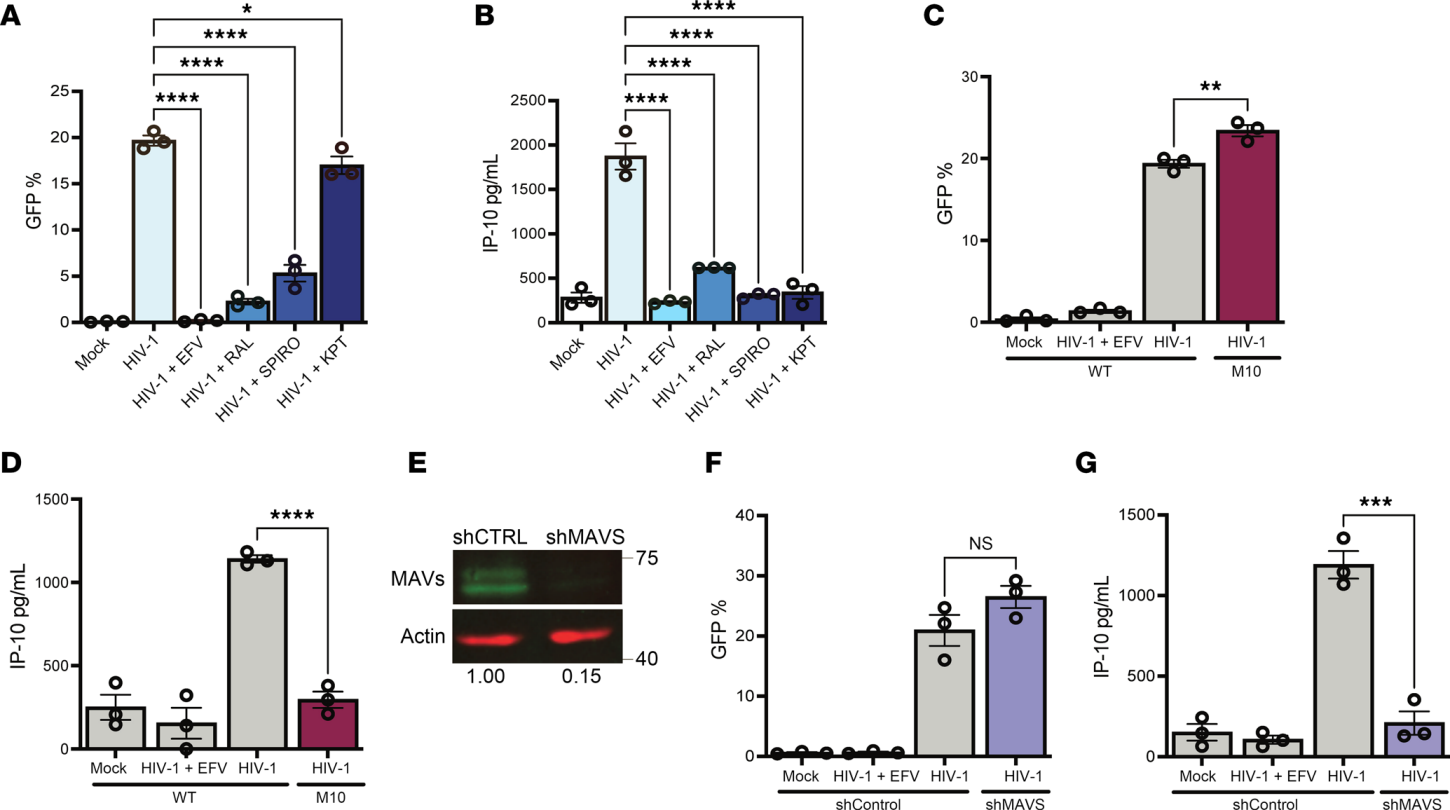
MAVS-mediated sensing of cytoplasmic icRNA triggers an innate immune response. THP-1/PMA macrophages were infected with LaiΔenv GFP/G (MOI 2) in the presence or absence of (1 μM), raltegravir (RAL; 30 μM), spironolactone (Spiro;100 nM), and KPT335 (KPT; 1 μM). Cells and culture supernatants were harvested at 3 dpi for (**A**) flow cytometry analysis to measure infection levels (%GFP^+^) and (**B**) ELISA to measure IP-10 secretion. (**C**) Infection levels and (**D**) IP-10 secretion in THP-1/PMA macrophages infected with either WT (LaiΔenvGFP/G) or HIV-1/M10 were determined at 3 dpi by flow cytometry and ELISA, respectively. (**E**) MAVS expression in THP1 cells transduced with shCTRL or shMAVS lentivectors was determined by Western blot analysis. (**F** and **G**) LaiΔenvGFP/G-infected THP-1/PMA macrophages and cell supernatants were harvested at 3 dpi for (**F**) flow cytometry analysis and (**G**) ELISA to measure infection establishment (%GFP^+^) and IP-10 secretion. Data are displayed as the means ± SEM, with each dot representing an independent experiment. Statistical significance was assessed via 1-way ANOVA with Dunnett’s multiple-comparison test (**A** and **B**) or unpaired *t* test (**C**, **D**, **F**, and **G**). *P < 0.05, **P < 0.01, ***P < 0.001, and ****P < 0.0001.

Previous work from our group and others identified that Rev/CRM1-mediated export of HIV-1 icRNA in MDMs and DCs was required for induction of MAVS-dependent innate immune responses, though the identity of the sensor or signaling pathway downstream of MAVS activation has remained unclear (29, 30). To further characterize this signaling pathway in a tractable system, we employed the Tohoku Hospital Pediatrics-1 (THP-1) monocytic cell line and differentiated cells with phorbol 12-myristate-13-acetate (PMA) to macrophage-like cells (37). THP-1/PMA macrophages were infected with vesicular stomatitis virus G glycoprotein**–**pseudotyped (VSV-G**–**pseudotyped) single-cycle HIV-1 encoding GFP as a reporter in place of nef (LaiΔenvGFP/G) (Figure 1A), and proinflammatory cytokine CXCL10 (IP-10) secretion in infected cultures was employed as a quantitative measure of infection-induced innate immune activation. Similar to findings in MDMs, establishment of productive infection in THP-1/PMA macrophages resulted in robust secretion of IP-10, which was abrogated in the presence of inhibitors that target reverse transcriptase (efavirenz [EFV]), integrase (raltegravir), or viral transcription (spironolactone) (38, 39) (Figure 1B). Furthermore, infection with HIV-1/M10 mutant (Figure 1C) that is incapable of exporting HIV-1 icRNA via the Rev/CRM1 pathway (31) or infection in the presence of CRM1 inhibitor (KPT330) also failed to induce IP-10 secretion (Figure 1, B and D). Knockdown of MAVS expression in THP-1/PMA macrophages (Figure 1E and Supplemental Figure 1, A–E; supplemental material available online with this article; https://doi.org/10.1172/JCI187663DS1) significantly decreased IP-10 secretion in virus-infected cells (Figure 1, F and G). Collectively, these findings suggest that sensing of a posttranscriptional step of the HIV-1 replication cycle induces innate immune responses in THP-1/PMA macrophages, recapitulating previous findings in HIV-1–infected MDMs and DCs (29, 30).

### MDA5 is required for HIV-1–induced innate immune responses in macrophages.

**Figure 2:**
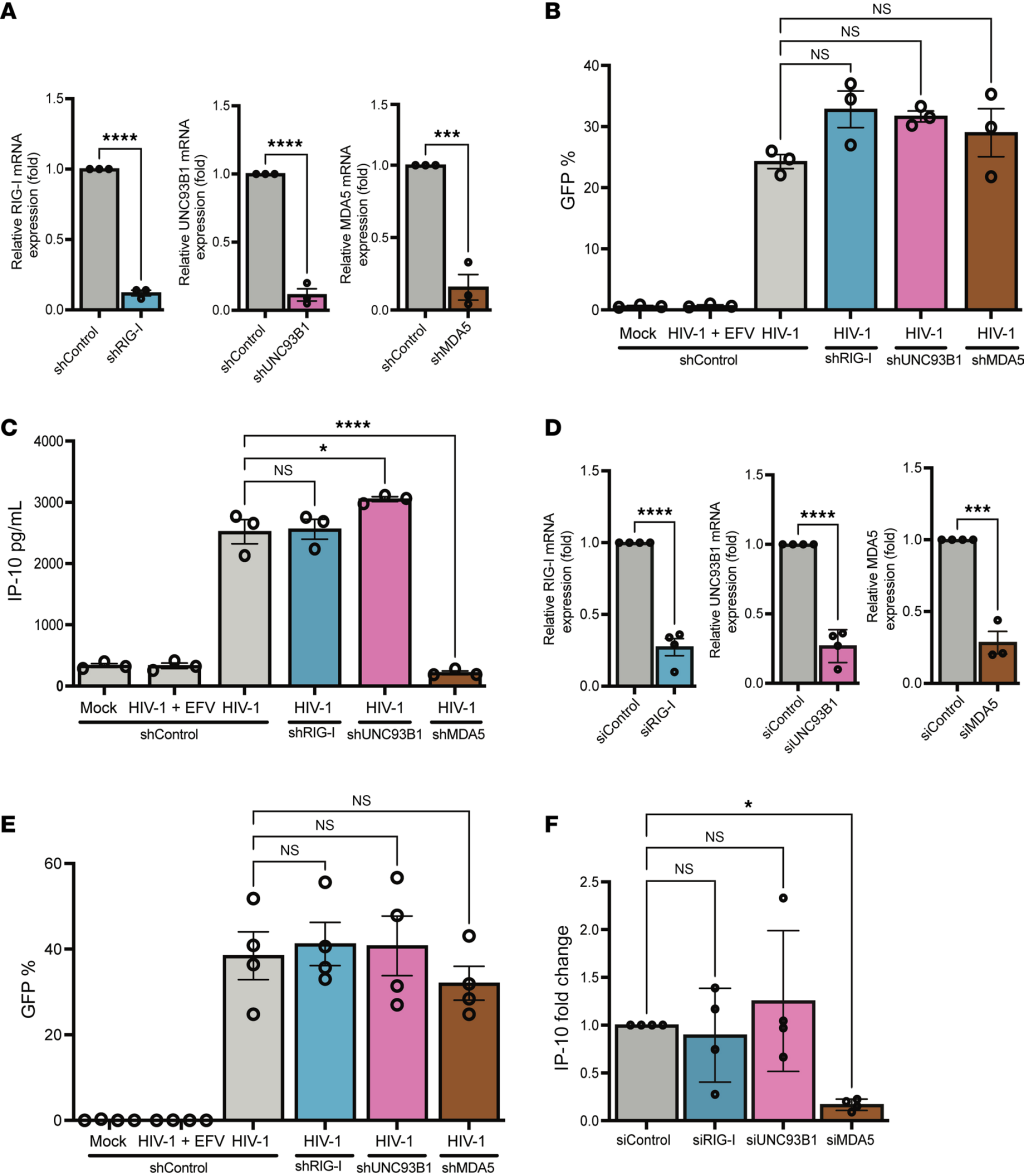
MDA5 is required for HIV-1–induced innate immune response in macrophages. (**A**) RIG-I, UNC93B1, and MDA5 expression in THP-1 cells transduced with RIG-I, UNC93B1, MDA5, or control shRNA-expressing lentivectors was quantified via RT-qPCR. (**B**) THP-1/PMA knockdown cell lines were infected with LaiΔenvGFP/G at MOI 2 and harvested at 3 dpi for infection establishment (%GFP^+^) via flow cytometry. (**C**) Supernatants from infected THP-1/PMA cells were used to assess IP-10 secretion via ELISA. (**D**) MDMs were transfected with siRNA targeting RIG-I, UNC93B1, and MDA5 for 2 days, and knockdown of RIG-I, UNC93B1, and MDA5 expression was assessed via RT-qPCR. MDMs were infected with LaiΔenvGFP/G at MOI 1 in the presence of dNs and harvested at 2 dpi for analysis of (**E**) infection efficiency via flow cytometry and (**F**) IP-10 mRNA expression via RT-qPCR. Data are displayed as the means ± SEM, with each dot representing an independent experiment (**A**–**C**) or cells from an independent donor (**D**–**F**). Statistical significance was assessed via unpaired *t* test (**A** and **D**) or 1-way ANOVA with Dunnett’s multiple comparisons (**B**, **C**, **E**, and **F**). *P < 0.05, ***P < 0.001, and ****P < 0.0001.

In mammalian cells, detection of viral RNAs in the cytosol and endosomes is surveilled by nucleic acid receptors. To identify the nucleic acid–sensing mechanism required for detection of HIV-1 icRNA, we generated THP-1 cell lines with stable knockdown of RIG-I (sensor of short, blunt-ended dsRNAs with an uncapped 5′ triphosphate group), MDA5 (sensor of long dsRNAs), or UNC93B1 (chaperone protein required for endosomal TLR3/7/8 function) expression in THP-1 cells via lentiviral transduction of shRNA (40). Knockdown efficiency was measured at the mRNA level via reverse transcription–quantitative PCR (RT-qPCR) (Figure 2A), while decrease in RLR or endosomal TLR activity was functionally validated by measuring IP-10 secretion in knockdown cells in response to RLR or TLR agonists (Supplemental Figure 1, F–J). While single-cycle HIV-1 (LaiΔenvGFP/G) infection of THP-1/PMA macrophages with reduced expression of RIG-I, MDA5, or UNC93B1 was unaffected compared with control shRNA-expressing cells (Figure 2B), we found that only MDA5, but not RIG-1 or UNC93B1, knockdown resulted in significant downregulation of HIV-1 icRNA-induced IP-10 production (Figure 2C). We next recapitulated these findings in primary MDMs. MDMs were transfected with pooled siRNAs against RIG-I, MDA5, or UNC93B1, and reduction in mRNA expression of targets was verified via RT-qPCR (Figure 2D). Upon infection of MDMs with LaiΔenvGFP/G (Figure 2E), we observed downregulation of IP-10 and IFN-β mRNA expression upon MDA5 knockdown, but not upon knockdown of either RIG-I or UNC93B1 (Figure 2F and Supplemental Figure 1K). Similar to findings with single-cycle HIV-1 (LaiΔenvGFP/G), knockdown of MDA5 also significantly attenuated IP-10 secretion from MDMs infected with replication-competent, CCR5-tropic Lai-YU2env viral isolate (Supplemental Figure 3, A–E). Taken together, these results suggest that MDA5 is required for induction of innate immune response upon cytoplasmic HIV-1 icRNA expression in MDMs.

### MDA5 recognizes unspliced HIV-1 RNA in the cytoplasm.

**Figure 3:**
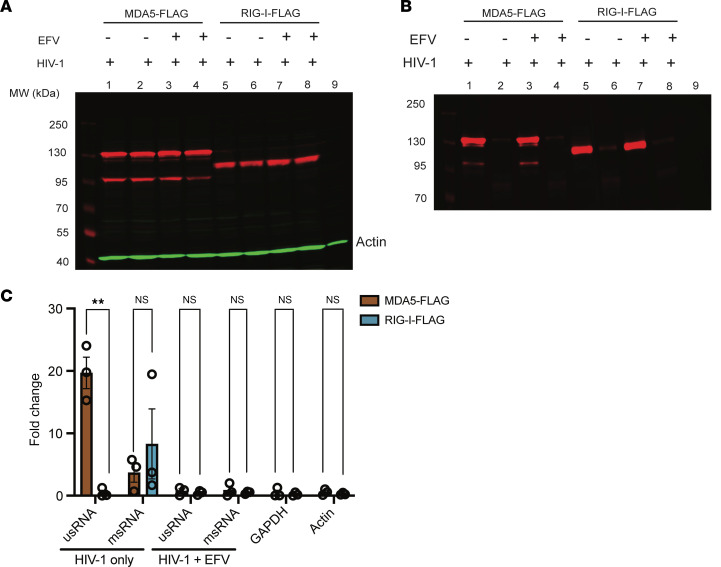
MDA5 recognizes unspliced HIV-1 RNA in the cytoplasm. HEK293T cells were infected with LaiΔenvGFP/G at MOI 1 and transfected with either MDA5-Flag or RIG-I–Flag at 24 hours after infection. Cytoplasmic fractions were immunoprecipitated with either anti-Flag mAb or IgG-coated beads. (**A**) Input and (**B**) IP lysates were run on Western blot to ensure equivalent levels of transfection and immunoprecipitation among conditions. (**C**) RT-qPCR analysis for HIV-1 usRNA, HIV-1 msRNA, GAPDH, or actin mRNA in transfected and infected HEK293T cells in the presence or absence of EFV. Fold enrichment of immunoprecipitated RNA reported as RNA transcripts detected from each IP condition using anti-Flag mAb or control IgG compared with the input amount. Data are displayed as means ± SEM, with each dot representing a different experiment. Statistical significance was assessed via unpaired *t* tests (**C**). **P < 0.01.

To investigate the interaction of MDA5 with HIV-1 icRNA, HEK293T cells were infected with LaiΔenv GFP/G in the presence or absence of EFV and then transfected with plasmids expressing either Flag epitope–tagged MDA5 or RIG-I. To ensure equivalent expression levels of MDA5 and RIG-I, cells were transfected with varying amounts of expression plasmids to optimize transfection conditions (Supplemental Figure 1L). RNA–protein interactions were stabilized in situ via UV cross-linking prior to fractionation of cell lysates to nuclear and cytoplasmic fractions, and immunoprecipitation of ribonucleoprotein particles (RNPs). Immunoblots confirmed equivalent MDA5 and RIG-I expression in cytoplasmic fractions (Figure 3A) and immunoprecipitation by anti-Flag mAb (Figure 3B). Immunoprecipitated RNA was quantified via RT-qPCR with primers complementary to *gag* (unspliced RNA [usRNA]), *tat-rev* (multiply spliced RNA [msRNA]), *GAPDH*, or *actin*. In concordance with the robust functional attenuation of HIV-1 icRNA sensing upon knockdown of MDA5 expression, HIV-1 usRNA was substantially enriched in RNPs immunoprecipitated with anti-Flag mAb in cells expressing MDA5-Flag (mean ~20-fold enrichment for MDA5), but not in those expressing RIG-I–Flag or control IgG immunoprecipitates (Figure 3, B and C). In contrast, no significant differences were observed in the level of msRNA coimmunoprecipitated with MDA5 or RIG-I. Specificity of HIV-1 usRNA immunoprecipitation by MDA5 was confirmed by the absence of GAPDH or actin RNAs in anti-Flag or IgG immunoprecipitates (Figure 3, B and C). These findings are in agreement with recently published studies that demonstrate the ability of MDA5 to specifically recognize HIV-1 usRNA in virus-infected dendritic cells (35). Taken together, these results suggest that MDA5 specifically interacts with HIV-1 icRNA and that MDA5 sensing of HIV-1 icRNA triggers innate immune activation in macrophages.

### IRF5 is a mediator of HIV-1–induced IP-10 production in macrophages.

**Figure 4:**
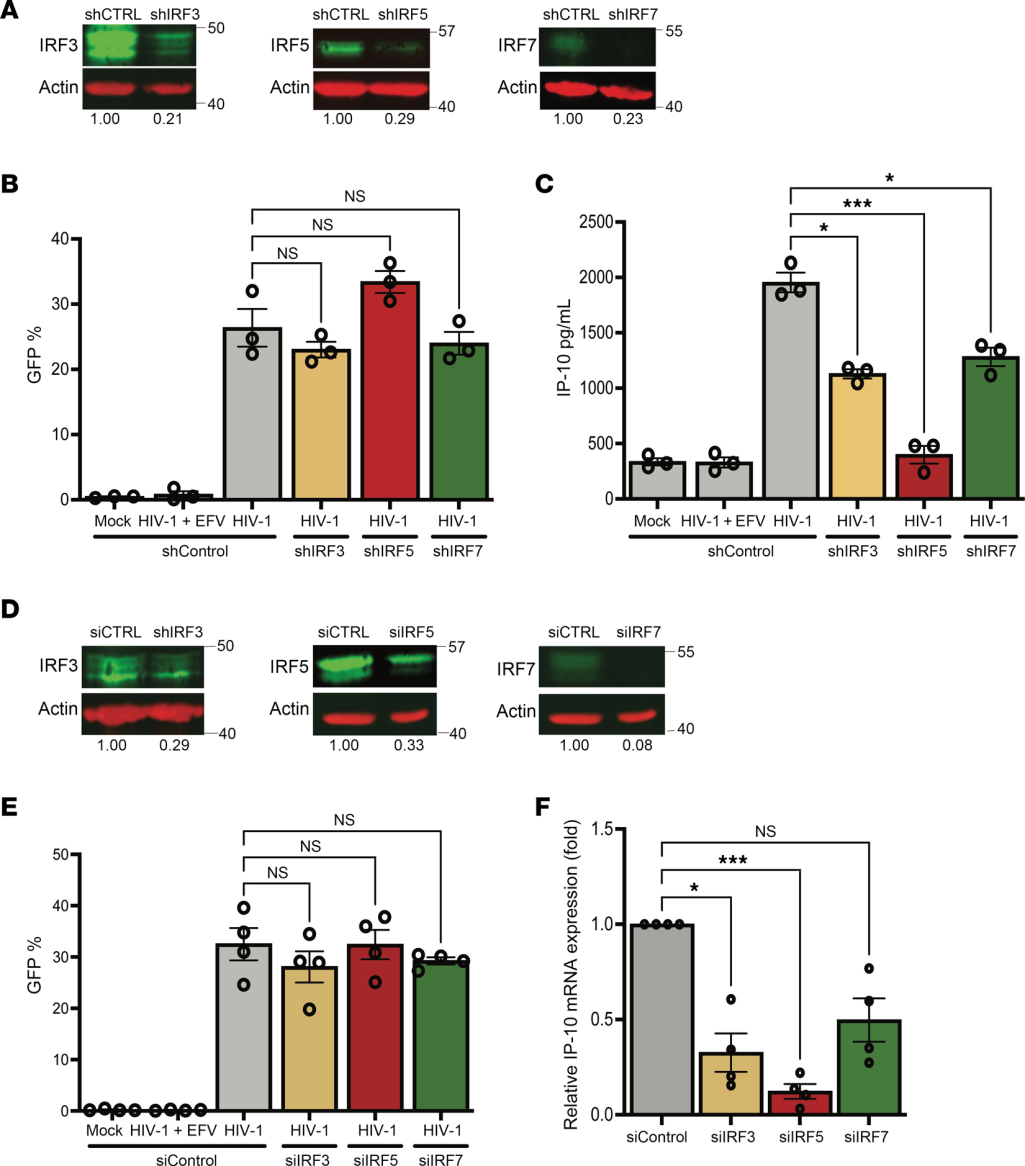
IRF5 is necessary for HIV-1 icRNA-induced IP-10 expression in macrophages. (**A**) IRF3, IRF5, and IRF7 expression in THP-1 cells transduced with IRF3, IRF5, IRF7, or control shRNA lentivectors was quantified via Western blot analysis and normalized to control shRNA transduced cells. (**B** and **C**) THP1/PMA macrophages were infected with LaiΔenvGFP/G at MOI 2, and cells and supernatants were harvested at 3 dpi for analysis via (**B**) flow cytometry to assess infection levels and (**C**) ELISA for IP-10 secretion. (**D**) Expression of IRFs in MDMs transfected with siRNA against IRF3, IRF5, or IRF7 mRNA was assessed via RT-qPCR. (**E** and **F**) MDMs were infected with LaiΔenvGFP/G at MOI 1 in the presence of dNs and harvested at 2 dpi for analysis of (**E**) infection efficiency via flow cytometry and (**F**) IP-10 expression via RT-qPCR. Data are displayed as the means ± SEM, with each dot representing a different donor. Statistical significance was assessed via 1-way ANOVA with Dunnett’s multiple comparisons (**B**, **C**, **E**, and **F**). *P < 0.05 and ***P < 0.001.

Upon sensing of viral RNAs, caspase activation and recruitment domain–dependent (CARD-dependent) interactions of MDA5 with MAVS lead to MAVS oligomerization and activation of downstream transcription factors, including NF-κB and IRFs (41). Within the IRF family of transcription factors, IRF3, IRF5, and IRF7 have well-described roles in mediating antiviral and inflammatory responses downstream of diverse viral infections (42). To characterize the roles of these IRFs in mediating HIV-1 icRNA-induced innate immune response in macrophages, we generated THP-1 cells with stable knockdown of IRF3, IRF5, or IRF7 expression using lentiviral shRNA transduction. We confirmed knockdown of these IRFs via Western blot (Figure 4A) and RT-qPCR (Supplemental Figure 2, A–C) and observed similar efficiency of decrease in IRF3, IRF5, or IRF7 expression. Functional knockdown was characterized by measuring response to 3p-hpRNA (RIG-I agonist) or LPS (TLR4 agonist) treatment. Decreased IRF3, IRF5, or IRF7 expression in THP-1/PMA macrophages led to attenuated responses to both 3p-hpRNA and LPS (Supplemental Figure 2, D and E). THP-1/PMA macrophages were infected with LaiΔenvGFP/G, and infection efficiency was assessed via flow cytometry at 3 days postinfection (dpi). We found there was no significant effect of IRF3, IRF5, or IRF7 knockdown on HIV-1 infection (Figure 4B). Culture supernatants were harvested and analyzed for IP-10 production via ELISA. While knockdown of IRF5 expression resulted in robust downregulation of HIV-1 icRNA-induced IP-10 production, decrease in IRF3 or IRF7 expression led to a modest though significant attenuation of IP-10 secretion (Figure 4C). Interestingly, requirement of IRFs for IP-10 secretion was dependent on the nature of viral PAMPs and the RLR sensing pathway, as IRF3, but not IRF5 or IRF7, was selectively required for Sendai virus–induced RIG-I–dependent IP-10 secretion (Supplemental Figure 2, F and G). To confirm these findings in primary MDMs, expression of IRF3, IRF5, and IRF7 was knocked down via transient transfection with pooled siRNAs (Figure 4D, Supplemental Figure 2, H–J, and Supplemental Figure 3, F–H) prior to infection with LaiΔenvGFP/G or with replication-competent Lai-YU2*env* virus. While knockdown of IRF3, IRF5, or IRF7 expression had no impact on the efficiency of infection establishment (Figure 4E and Supplemental Figure 3I), we observed significant downregulation in IP-10 expression upon IRF5 or IRF3 knockdown and a trend toward significance upon knockdown of IRF7 expression in HIV-infected MDMs (Figure 4F and Supplemental Figure 3J). In contrast to IP-10, expression of IFN-β was robustly attenuated by knockdown of IRF3, IRF5, or IRF7 expression in HIV-1–infected MDMs (Supplemental Figure 2K), suggesting a selective and nonredundant role of IRF5 in mediating a proinflammatory response to HIV-1 infection in macrophages.

### TRAF6 and IKK-β are required for IP-10 production in HIV-1–infected macrophages.

**Figure 5:**
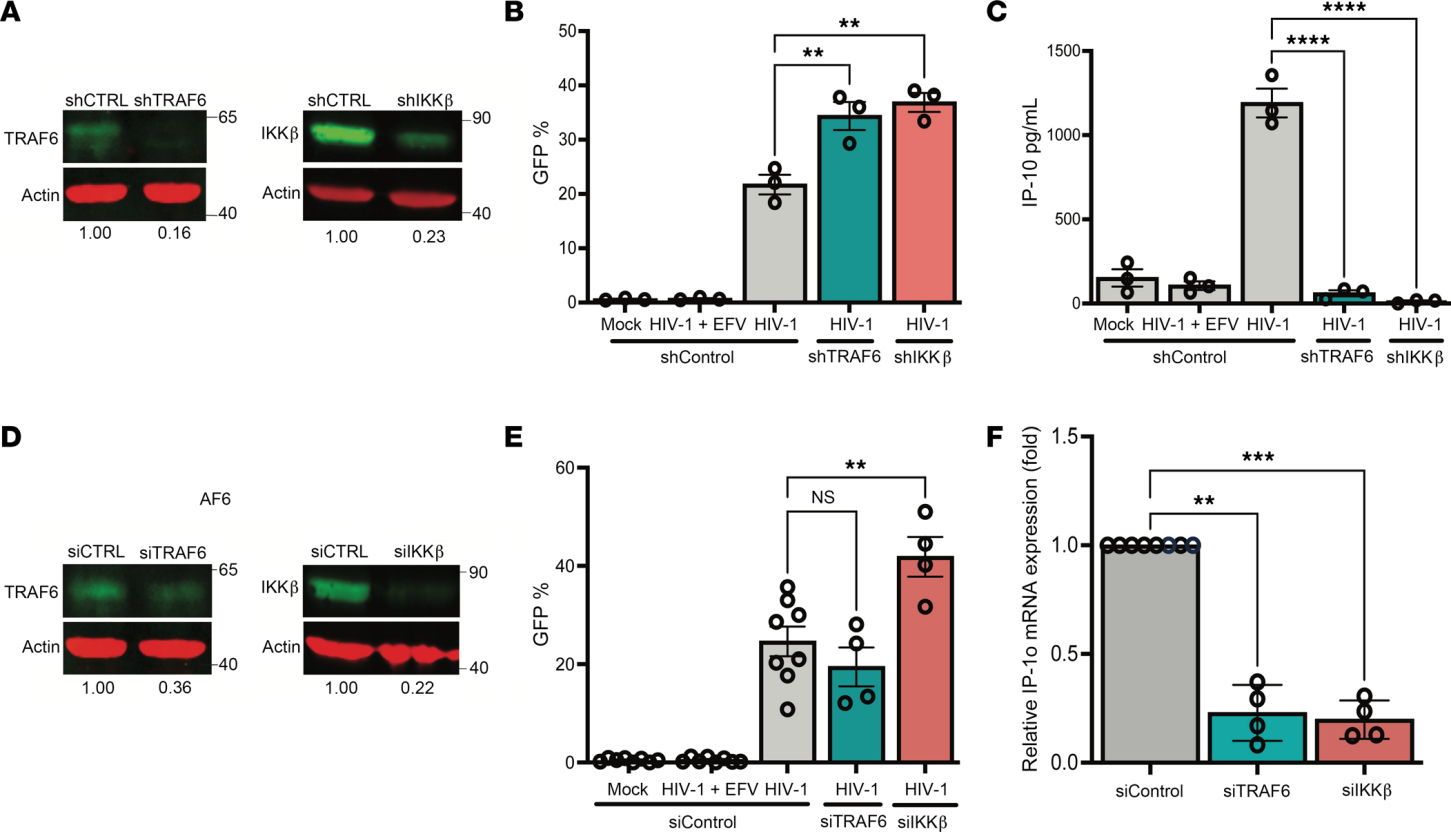
TRAF6 and IKK-β are required for HIV-1–induced IP-10 expression in macrophages. (**A**) TRAF6 or IKK-β expression in THP-1 cells transduced with TRAF6 or IKK-β shRNA lentivectors was quantified via Western blot analysis and normalized to control shRNA transduced cells. THP1/PMA macrophages infected with LaiΔenvGFP/G at MOI 2 and harvested at 3 dpi for analysis via (**B**) flow cytometry to assess infection levels and (**C**) ELISA to assess IP-10 secretion. (**D**) MDMs were transfected with siRNA against TRAF6 or IKK-β mRNA for 2 days, and knockdown of TRAF6 or IKK-β was assessed via Western blot and RT-qPCR. (**E** and **F**) MDMs infected with LaiΔenvGFP/G at MOI 1 in the presence of dNs and harvested at 2 dpi for analysis of (**E**) infection efficiency via flow cytometry and (**F**) IP-10 expression by RT-qPCR. Data are displayed as the means ± SEM, with each dot representing a separate experiment (**A**–**C**) with cells from independent donors (**D**–**F**). Statistical significance was assessed via 1-way ANOVA with Dunnett’s multiple comparisons (**B**, **C**, **E**, and **F**). **P < 0.01, ***P < 0.001, and ****P < 0.0001.

Upon sensing of viral RNA by MDA5 and activation of the MAVS signalosome, diverse kinases, including IKK-β, IKK-ε, and TBK1, and ubiquitin ligases, such as TRAF2, TRAF5, and TRAF6, are recruited and activated, which in turn posttranslationally modify IRFs and NF-κB to drive expression of antiviral genes and inflammatory cytokines (43, 44). It has previously been shown that the E3 ubiquitin ligase TRAF6 and serine kinase IKK-β are required for K63-linked polyubiquitination and phosphorylation events and IRF5 activation downstream of TLR and RLR sensing (45), though their requirement for HIV-1–induced type I IFN responses and IP-10 production has not been assessed. To determine whether IKK-β and TRAF6 are required for IP-10 production upon HIV-1 icRNA sensing in macrophages, we utilized lentiviral shRNA to generate stable TRAF6 or IKK-β knockdown THP-1 cell lines. Knockdown of gene expression of these factors was validated by Western blot and RT-qPCR (Figure 5A and Supplemental Figure 2, L and M). These knockdown cell lines were also validated functionally by measuring IP-10 secretion in response to RLR and TLR agonists such as 3p-hpRNA and LPS (Supplemental Figure 2, N and O). While knockdown of TRAF6 or IKK-β expression resulted in no difference in HIV-1 infection (Figure 5B), we observed a significant decrease in IP-10 production in HIV-1–infected THP-1/PMA macrophages (Figure 5C). In contrast, IP-10 secretion was only modestly attenuated in herpes simplex virus 1 (HSV-1) or Sendai virus–infected THP-1/PMA macrophages deficient for TRAF6 or IKK-β expression (Supplemental Figure 2, P and Q). These results suggest that TRAF6 has a nonredundant role in the induction of IP-10 expression in response to HIV-1 icRNA sensing by MDA5. We next sought to determine the roles of TRAF6 and IKKβ in transducing signals downstream of HIV-1 icRNA sensing in primary MDMs. MDMs were transfected with pooled siRNAs against TRAF6 or IKK-β prior to infection with LaiΔenvGFP/G or replication-competent HIV-1/Lai-YU2*env*. Western blot and RT-qPCR analysis confirmed robust knockdown efficiency of both TRAF6 and IKK-β in MDMs (Figure 5D; Supplemental Figure 2, R and S; and Supplemental Figure 3, K and L). While knockdown of TRAF6 or IKK-β expression did not attenuate HIV infection (Figure 5E and Supplemental Figure 3M), there was a robust reduction in HIV-1 icRNA-induced IP-10 and IFN-β mRNA expression upon knockdown of TRAF6 or IKK-β expression in MDMs (Figure 5F, Supplemental Figure 2T, and Supplemental Figure 3N), indicating an important role of TRAF6 and IKK-β in MDA5/MAVS-dependent activation of IRF5 for induction of IP-10 and type I IFN responses in HIV-1–infected macrophages.

### HIV-1 infection results in nuclear translocation of IRF5 in THP-1/PMA macrophages.

**Figure 6:**
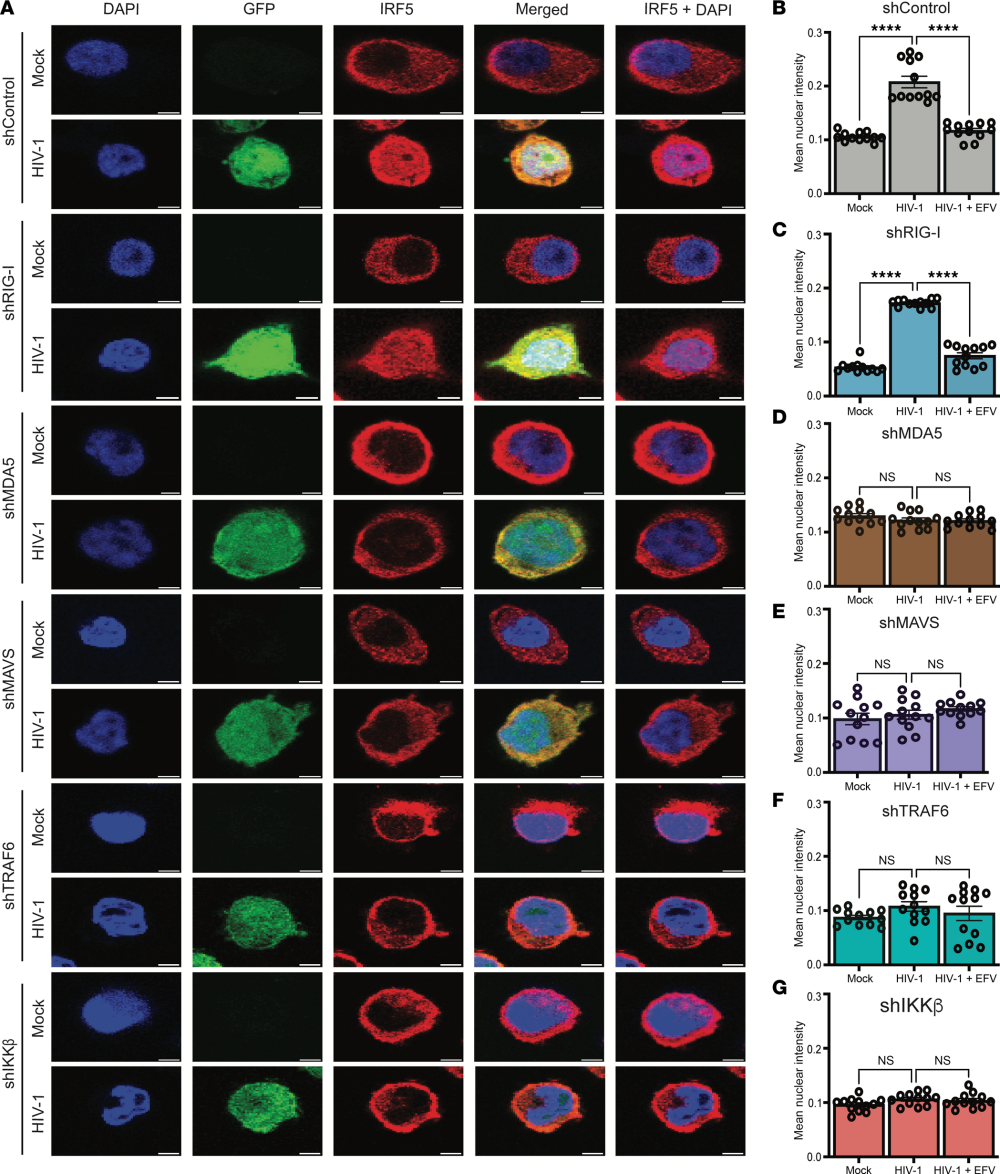
MDA5, MAVS, TRAF6, and IKK-β are required for HIV-1 icRNA-induced nuclear localization of IRF5 in macrophages. (**A**) THP-1/PMA macrophages infected with LaiΔenvGFP/G at MOI 2 and harvested at 3 dpi for analysis via confocal microscopy to assess changes in IRF5 localization. Representative images are shown. Scale bar: 5 mm. (**B**–**F**) Fluorescence microscopy images were quantified via CellProfiler to assess mean pixel intensity of IRF5 staining that colocalized with DAPI (mean nuclear intensity). Images from 3 independent infection experiments were analyzed and quantified, with each dot representing a field containing approximately 50–150 cells. Statistical significance was assessed via Kruskal-Wallis test with Dunn’s multiple-comparison analysis (**B**–**G**). ****P < 0.0001.

Since IRF5 is an ISG (46), and HIV-1 infection of macrophages induces type I IFN responses, we next sought to determine if IRF5 activation in HIV-infected MDMs occurs directly downstream of MDA5/MAVS sensing of HIV icRNA in a cell-intrinsic manner in infected cells or requires activation in bystander uninfected cells. IRF5 exists in an inactive form in the cytosol in unstimulated cells, posttranslational modifications of which result in its translocation to the nucleus (47). To visualize IRF5 activation in response to HIV-1 infection in macrophages, we utilized immunofluorescence and confocal microscopy. THP-1/PMA macrophages were infected with LaiΔenvGFP/G and stained for intracellular localization of IRF5 expression on coverslips at 3 dpi. DAPI was utilized as a nuclear stain, and HIV-infected cells were distinguished by GFP positivity. HIV-1 (WT) infection of THP-1/PMA macrophages resulted in nuclear translocation of IRF5 from cytoplasm in infected cells (Figure 6A), which was not observed upon infection with HIV-1/M10 mutant (Supplemental Figure 5A). To quantify nuclear translocation of IRF5, we assessed the mean signal intensity of nuclear IRF5 using CellProfiler (Supplemental Figure 4). In HIV-1 (WT)–infected, GFP^+^ THP-1/PMA macrophages, we observed a marked increase in nuclear IRF5 staining that was blocked upon pretreatment with EFV or upon infection with HIV-1/M10 mutant virus (Figure 6B and Supplemental Figure 5A). Furthermore, nuclear localization of IRF5 was selectively enhanced in GFP^+^ cells with HIV WT infection (Supplemental Figure 5B), suggesting that cell-intrinsic HIV-1 icRNA sensing activates IRF5. Importantly, nuclear IRF5 localization in HIV-1 (WT)–infected cells was attenuated upon depletion of MDA5, MAVS, IKK-β, or TRAF6 expression (Figure 6, D–G), but not RIG-I expression (Figure 6C). These results indicate that MDA5, MAVS, IKK-β, and TRAF6 are required for IRF5 activation and nuclear localization directly downstream of HIV-1 icRNA sensing in macrophages.

### Macrophages and monocytes isolated from older donors exhibit elevated levels of IRF5 expression.

**Figure 7:**
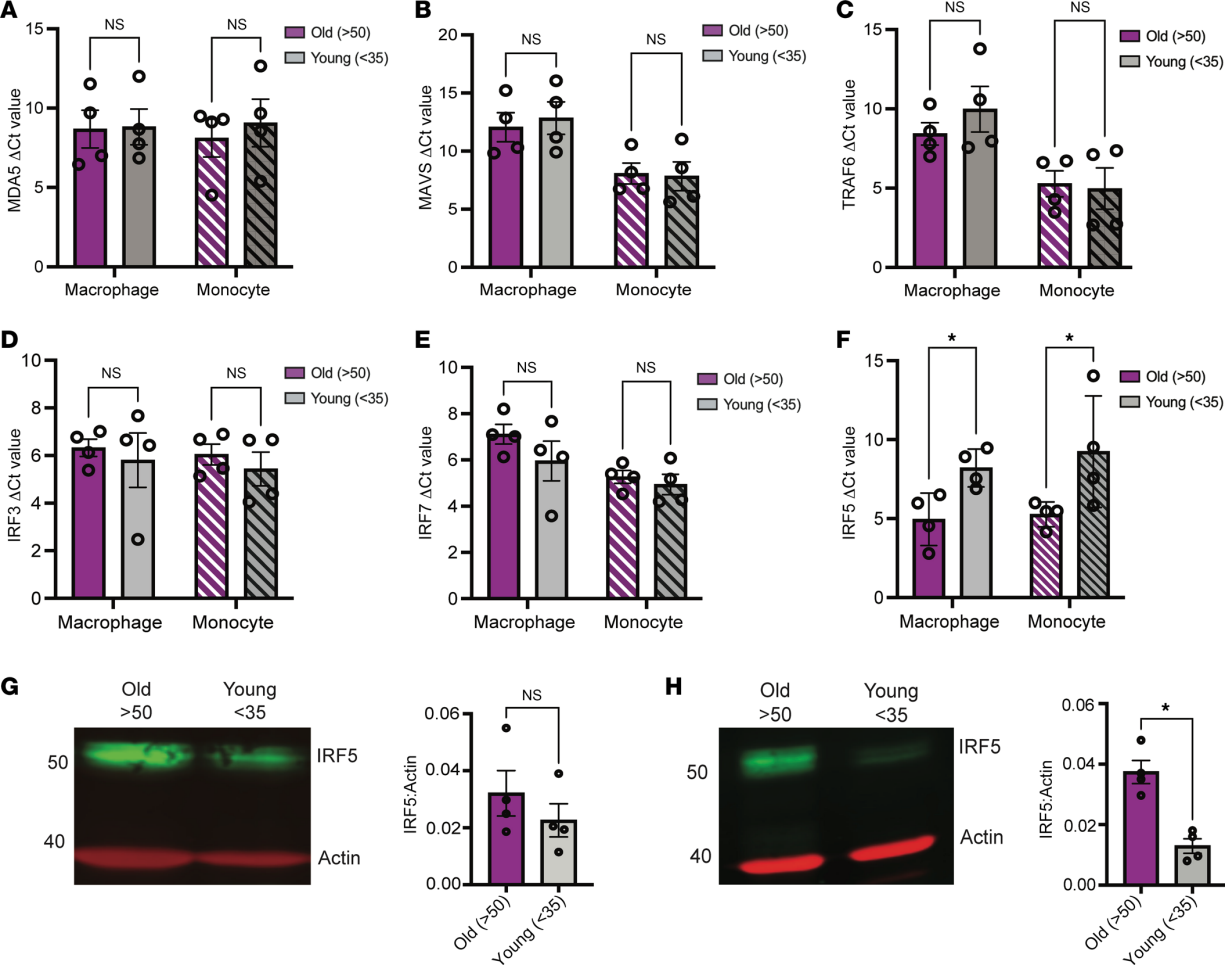
MDMs and monocytes isolated from older individuals exhibit higher levels of IRF5 expression. (**A**–**F**) Constitutive mRNA expression of (**A**) MDA5, (**B**) MAVS, (**C**) TRAF6, (**D**) IRF3, (**E**) IRF7, and (**F**) IRF5 in MDMs and monocytes was assessed via RT-qPCR. (**G** and **H**) Western blot analysis for constitutive IRF5 expression in MDMs and monocytes. Data are displayed as mean ± SEM, with each dot representing a donor. Statistical significance was assessed via unpaired *t* tests (**A**–**H**). *P < 0.05.

Several cohort studies have demonstrated that older PWH experience comorbidities and age-associated disease at a greater level compared with age-matched people without HIV (48–50). Despite suppression of viremia, HIV-1 RNA and DNA can persist in long-lived cells such as macrophages and may contribute to the development of comorbidities in PWH (51–57). We sought to determine whether macrophages isolated from older compared with younger individuals exhibit a greater inflammatory response to HIV-1 icRNA. To test this hypothesis, we utilized samples from 2 cohorts, 1 from the HIV/Aging cohort at Boston Medical Center (BMC) (Supplemental Table 1) and the other from New York Biologics Blood Center (Supplemental Table 2). Donors were stratified by age as older (>50 yrs) or younger (18–35 yrs). PBMCs were isolated from whole blood samples from people without HIV, and CD14^+^ monocytes were collected via positive selection on the day of sample collection and differentiated to MDMs. To investigate basal expression differences in factors involved in sensing of HIV-1 icRNA, we quantified expression levels of MDA5, MAVS, TRAF6, and IRF3, IRF5, or IRF7 in CD14^+^ monocytes and MDMs from younger and older donors. We did not observe any age-associated differences in the expression of MDA5, MAVS, TRAF6, IRF3, or IRF7 expression in peripheral blood CD14^+^ monocytes and MDMs derived from PBMCs from the NY Biologics Blood Center (Figure 7, A–E). Interestingly, IRF5 expression was constitutively elevated in CD14^+^ monocytes and MDMs from older individuals (Figure 7F). We validated this finding by measuring IRF5 protein level via Western blot and found that IRF5 protein expression was significantly elevated in older monocytes and trended to higher expression in older macrophages (Figure 7, G and H).

### Innate immune sensing of HIV-1 infection in macrophages is impacted by age.

**Figure 8:**
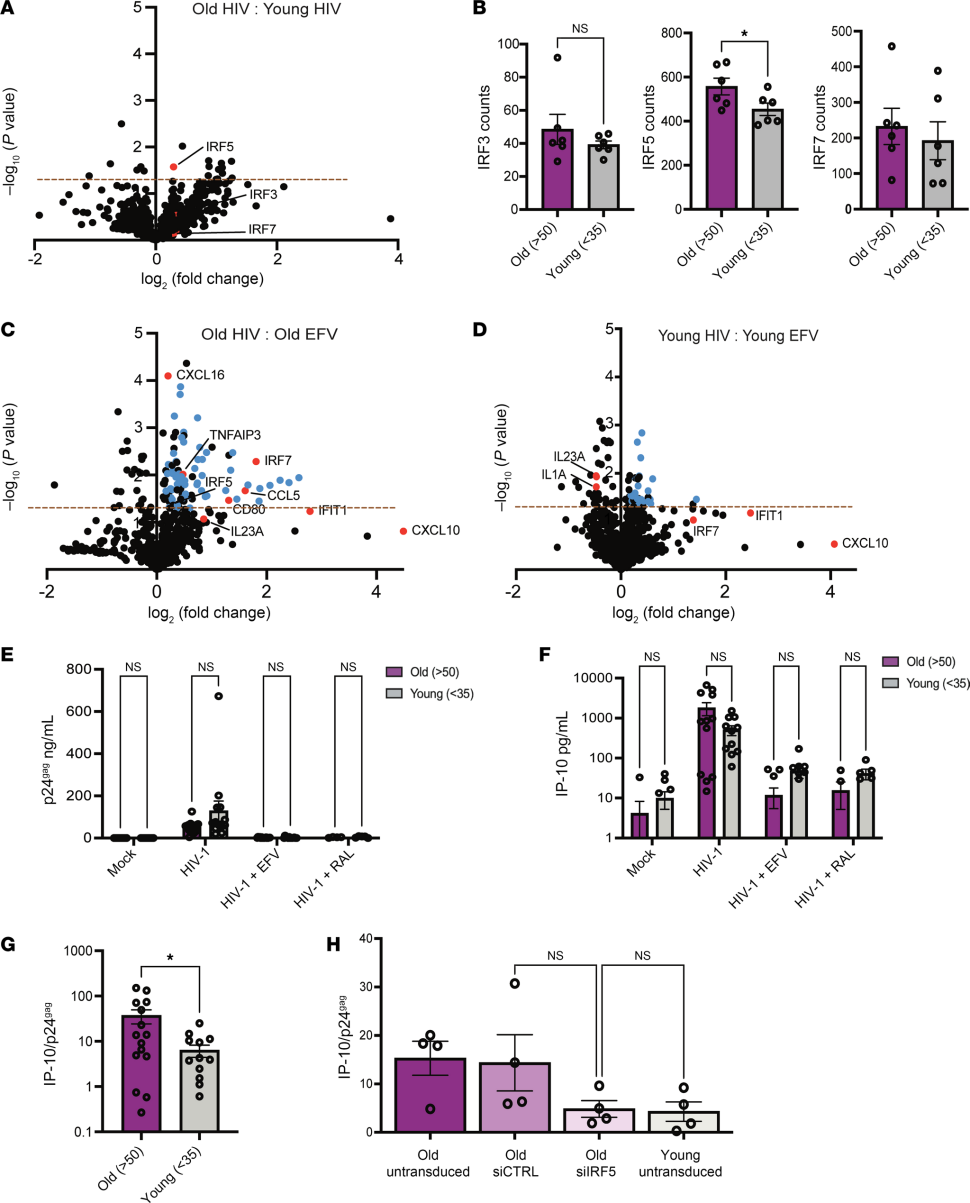
MDMs isolated from older donors exhibit higher levels of HIV-1–induced immune activation. (**A**) RNA isolated from LaiΔenvGFP/G-infected MDMs (MOI 2) was analyzed via Nanostring nCounter using the Myeloid Innate Immunity V2 panel. Baseline expression of the gene panel was calculated using nSolver and plotted as a ratio of old (HIV) versus young (HIV) with IRFs (red) highlighted. The dashed line represents a *P* value of 0.05. (**B**) Raw count values for IRF3, IRF5, and IRF7 were plotted to assess differences. (**C** and **D**) Fold changes in innate immune gene expression in HIV-infected MDMs with or without EFV were plotted. Data from MDMs from (**C**) older (>50 yo) and (**D**) younger (<35 yo) donors are shown. Differentially expressed ISGs (blue) and IRFs/IRF targets (red) are highlighted. The dashed line represents a *P* value of 0.05. (**E** and **F**) Supernatants were analyzed for (**E**) p24gag production and (**F**) IP-10 levels via ELISA. (**G**) IP-10 levels were normalized to those of p24gag for each donor. (**H**) MDMs were transfected with either control or IRF5 targeting siRNA prior to infection with LaiΔenvGFP/G. IP-10 and p24gag secretion was measured at 3 dpi. Data are represented as mean ± SEM, with each dot representing an individual donor (**B** and **E**–**G**) or with each dot representing a target gene (**A**, **C**, and **D**). Significance was assessed via unpaired 2-tailed *t* test (**A**, **B**, and **E**–**G**), paired 2-tailed *t* test (**C** and **D**), or 1-way ANOVA with Tukey’s multiple-comparison test (**H**). *P < 0.05.

Since IRF5 expression was elevated in macrophages from older donors, we next sought to assess age-related differences in innate immune response to HIV-1 infection. Total RNA was extracted from uninfected and HIV-infected MDMs from 12 donors from the BMC HIV/Aging cohort, and mRNA expression profiles were analyzed via Nanostring using the Myeloid Innate Immunity Panel consisting of 730 target genes. The samples were selected to ensure the same number of older/younger donors and equal numbers of male/female individuals within each group. We first compared differences in these myeloid innate immune genes at the baseline level in uninfected older and younger MDMs. We observed elevated mRNA levels of several IRF5 target genes including CCL21, IP-10, IL-6, and IL12A/B in older MDMs (Supplemental Figure 6A). We also observed slightly elevated expression of IRF5 in MDMs from older donors but did not observe this trend with IRF3 and IRF7 (Supplemental Figure 6B). We repeated this analysis using the virus only conditions and found that IRF5, but not IRF3 or IRF7, was significantly upregulated in HIV-1–infected MDMs isolated from older donors (Figure 8, A and B) despite similar levels of infection in vitro (Supplemental Figure 6C). We next sought to assess age-related differences in induction of the innate immune responses in older and younger MDMs upon HIV-1 infection by comparing the fold change in innate immune gene expression in the HIV-1–infected samples to the EFV control within each age group. We found that the number of upregulated genes and the extent of induction of innate immune gene expression varied based on age. MDMs from older donors demonstrated approximately 97 significantly upregulated genes compared with 42 genes from younger donors (Figure 8C). These upregulated genes were primarily ISGs, including IRF5 and IRF7, as well as several known IRF5 target genes (Figure 8C), including CCL5, CXCL16, and IL23A, suggesting that MDMs from older individuals display an enhanced IRF5-dependent innate immune response to HIV-1 infection. Additionally, while we observed that IP-10 was upregulated in both older and younger MDMs upon HIV-1 infection, the extent of upregulation was greater in older MDMs (Figure 8, C and D). Surprisingly, we observed no significant induction of IRF5-regulated target gene expression in MDMs from younger donors upon HIV-1 infection (Figure 8D). Instead, expression of IRF5-regulated genes IL1A and IL23A was significantly downregulated in response to HIV-1 infection in MDMs from younger donors (Figure 8D).

Interestingly, while there was no significant difference in extent of HIV-1 infection in MDMs derived from younger or older MDMs (Figure 8E and Supplemental Figure 7A), IP-10 production in HIV-infected older MDMs was higher than that observed with HIV-infected MDMs from younger donors (Figure 8F and Supplemental Figure 7B). The difference was evident even when IP-10 secretion levels were normalized to HIV-1 infection levels across donors to account for donor-to-donor variation in infection establishment (Figure 8G). In contrast, no significant age-associated differences in IP-10 production were observed upon stimulation with 3p-hp RNA (RIG-I agonist) or LPS (TLR4 agonist) in MDMs (Supplemental Figure 7, C–F), suggesting an HIV-specific enhanced innate immune response in macrophages from older donors. Finally, knockdown of IRF5 expression in older MDMs suppressed HIV infection–induced IP-10 secretion to levels observed in younger MDMs (Figure 8H). Taken together, these results indicate that the inflammatory response induced upon MDA5 sensing of HIV-1 icRNA expression in MDMs is impacted by age and cell-intrinsic IRF5 expression. Importantly, constitutively elevated IRF5 expression in macrophages from older donors might contribute to the heightened proinflammatory state and accelerated course of clinical disease in older PWH.

## Discussion

In this study, we highlight an important, nonredundant role of MDA5 in sensing of HIV-1 icRNA in macrophages and induction of type I IFN responses. These findings were corroborated in a recent study that showed MDA5-mediated sensing of HIV-1 icRNA in dendritic cells (35). Though the specific motif within HIV-1 icRNA that is recognized by MDA5 is currently unclear, sensing of non-self RNA by RLR family members is dependent on unique invariant features. For instance, discrimination between cellular and viral dsRNA by RIG-I is based on 5′ triphosphate motifs and blunt-ended RNA duplex structure of less than 100 bp in length, though size requirement can be variable (58). Though previous studies suggest that transfected HIV-1 RNA can be sensed by RIG-I, it is unclear whether this mechanism is conserved in infection models (59). Importantly, in our studies, knockdown of RIG-I expression in macrophages had no impact on HIV-1 icRNA-induced type I IFN responses. However, MDA5 does not require terminal 5′′ triphosphates for ligand recognition, but rather preferentially recognizes long dsRNAs (>500 bp) and utilizes length specificity to distinguish self from non-self dsRNAs (60, 61). Ligand binding nucleates MDA5 filaments on dsRNA and subsequent recruitment and induction of MAVS filament formation in a CARD-dependent manner (62).

Our results demonstrate specific immunoprecipitation of HIV-1 icRNA but not msRNA by MDA5 in virus-infected cells. It is plausible that MDA5 recognizes duplex regions of stem-loops (63, 64) or other uncharacterized RNA features uniquely existing in HIV icRNA. Further studies are warranted to elucidate the molecular mechanisms underlying MDA5 sensing of HIV icRNA. Interestingly, MDA5 has also been implicated in sensing of endogenous retroelement (ERE) 3′ UTR-containing dsRNAs generated either via bidirectional transcriptional mechanisms or upon cytoplasmic spillover of intron-retained ERE RNAs (65). While expression of ERE RNAs is enhanced due to transcriptional derepression in senescent or aging cells (66) or exacerbated due to HIV-1 infection (67, 68), contributions of ERE dsRNAs to innate immune activation in HIV-infected cells need further investigations. Additionally, while other studies have implicated TLR7 in sensing of HIV-1 genomic RNA in plasmacytoid dendritic cells (69), downregulation of UNC93B1 expression did not impact HIV-1 icRNA-induced IP-10 and IFN-β induction, suggesting the mechanism of HIV-1 RNA sensing varies in a cell-type–dependent manner and that MDA5 is required for sensing of intron-containing HIV-1 transcripts in macrophages.

This study also highlights an important role of IRF5 in the innate immune signaling pathway downstream of MDA5/MAVS-dependent HIV-1 icRNA sensing. While previous studies have implicated IRF3 and IRF7 in inducing expression of IFN-β and ISGs in HIV-1–infected cells (70), here we show that IRF5 also has an important role in inducing IFN-β and proinflammatory cytokine IP-10 expression in HIV-1–infected macrophages, suggesting functional redundancies in IRF requirements. This is consistent with previous findings that IRF5 does not bind to virus response elements in the promoter region of IFN-α, which highlights that IRF5 activation leads to the transcription of specific type I IFNs and ISGs (71). This IRF5 DNA binding specificity may also account for conflicting findings that IRF7, but not IRF5, is crucial for ISG15 induction in HIV-1–infected macrophages and DCs (35). We also found that the type of viral infection has an impact on IRF5 involvement in mediating IP-10 production. For instance, IRF5 knockdown attenuated IP-10 production in HSV-1 but not in Sendai virus–infected cells, highlighting that IRF5 activation might uniquely be dependent on the infecting virus and its replication intermediates.

Previous studies have shown that MAVS activation downstream of the RLR sensing pathways leads to recruitment of various E3 ubiquitin ligases (TRAFs) and cytosolic serine kinases, IKK and TBK1 (72, 73), for IFN-β induction. As opposed to the redundant roles of TRAF2/3 and TRAF6 for NF-κB and IRF3 activation and IFN-β induction downstream of Sendai virus infection (72), our results suggest a nonredundant role of TRAF6 in IRF5 activation downstream of HIV-1 icRNA sensing in macrophages. These findings align with the purported requirement for TRAF6-mediated K63-linked ubiquitination of IRF5 as an essential protein modification for IRF5 activation (44). Interestingly, knockdown of IKK-β abrogated IP-10 and IFN-β induction in HIV-1–infected macrophages, highlighting the essential role for additional posttranslational modifications of IRF5, such as phosphorylation (45, 47) downstream of HIV-1 icRNA sensing by MDA5 and MAVS. Taken together, we propose that HIV-1 icRNA sensing by MDA5 and MAVS activation leads to the recruitment and activation of TRAF6 and IKK-β, resulting in ubiquitination and phosphorylation and subsequent nuclear translocation of IRF5, contributing to IP-10 and IFN-β expression. IFN-β production might further induce IRF5 expression, thus exacerbating production of type I IFNs and proinflammatory cytokines.

As the population of individuals living with HIV increases in age, it is crucial to understand the mechanisms that contribute to higher levels of immune activation in older PWH (74). While duration of ART, sex, lifestyle, and comorbidities are all contributing factors, results described in this study propose a cell-intrinsic role of IRF5 in inducing innate immune activation in HIV-1–infected macrophages from older donors. We found that MDMs and monocytes from older donors in 2 distinct cohorts had higher baseline levels of IRF5 expression at the mRNA and protein level and consequently expressed elevated mRNA expression levels of several IRF5 target genes, including CCL21, IP-10, and IL-6. Upon HIV-1 infection of MDMs, we also observed a higher level of ISG induction in MDMs from older donors, including IRF5, as well as several known IRF5-responsive genes, such as CXCL16, CCL5, and CD80. IP-10 has been shown to be upregulated in the plasma of older individuals (75). Serum IL-6 levels also increase with age and have been associated with frailty and mortality (76–78) as well as age-related neurodegenerative diseases such as Alzheimer’s disease (79–81). Additionally, significantly higher serum levels of CXC16 were correlated with severe COVID-19 outcome, which is more common among older individuals (82, 83). Similar to previous studies that reported elevated IRF5 expression in monocytes from older donors upon RIG-I activation (84), MDA5/MAVS-mediated IRF5 nuclear translocation in HIV-1–infected macrophages and type I IFN-induced IRF5 expression might contribute to IRF5 hyperactivation.

Numerous studies have shown that macrophages are polarized to an inflammatory phenotype because of aging (85, 86). IRF5 expression polarizes macrophages toward an inflammatory state, and elevated IRF5 levels have been implicated in age-related conditions such as arthritis and glioma (87–90). Several studies have also shown that aging results in increased type I IFN secretion and upregulated IFNAR expression in immune cells (91, 92). Furthermore, IRF5 is an ISG, and persistent type I IFN signaling might result in upregulation of IRF5 expression, thus perpetuating the age-associated proinflammatory state. Since epigenomes of macrophages are highly plastic and susceptible to signals from the microenvironment (93, 94), putative drivers of aging-associated dysregulated IRF5 expression might include hypomethylated status of IRF5 promoter sequences or site-specific methylation changes resulting in altered accessibility of IRF5 at promoters of inflammatory genes. Several lines of evidence have indicated that aging is characterized by widespread promoter hypomethylation, which could extend to IRF5 promoters as well (95, 96). Further studies are warranted to investigate the interplay between cell-autonomous mechanisms of promoting inflammation and microenvironment cues. Taken together, these results suggest that enhanced levels of constitutive IRF5 expression in monocytes and macrophages from older donors and HIV infection–induced IRF5 activation may contribute to enhanced inflammatory responses in PWH and persistent expression of inflammaging-related genes.

IRF5 has been considered a promising therapeutic target for inflammatory diseases such as systemic lupus erythematosus and rheumatoid arthritis (87, 97). IRF5 function is cell-type specific and limited primarily to induce proinflammatory cytokines in response to infection (98, 99). Unlike other transcription factors, such as NF-κB, the downstream targets of IRF5 are more specific (89, 100), and incorporation of IRF5-targeting therapeutics in combination with ART could potentially reduce chronic inflammation and thus prevent the development of comorbidities associated with accelerated inflammaging in older PWH.

## Methods

### Sex as a biological variable.

Sex was not considered as a biological variable

### Plasmids.

Single-cycle HIV-1 encoding GFP in place of *nef* (LaiΔenv/GFP) and an HIV-1 Rev mutant (M10) deficient in CRM1 binding (LaiΔenv/GFP-M10) have been previously described (29, 101). Lentiviral vectors (pLKO.1) expressing shRNAs against IRF3, IRF5, IRF7, RIG-I, UNC93B1, MDA5, TRAF6, and IKK-β were purchased from Sigma-Aldrich (or constructed by ligating annealed double-stranded oligonucleotides into pLKO.1 using AgeI and EcoRI sites). HIV-1 packaging plasmid, psPAX2, and VSV-G expression plasmid, H-CMV-G, have been described previously (102).

### Cells.

HEK293T cells (ATCC) and TZM-bl (NIH AIDS Reagent Program; HRP 8129; contributed by John C. Kappes and Xiaoyun Wu, University of Alabama at Birmingham and Tranzyme Inc., Birmingham, Alabama, USA) were cultured in DMEM (Gibco) supplemented with 10% FBS (Gibco) and 1% penicillin/streptomycin (pen/strep) (Gibco). THP-1 cells (NIH AIDS Reagent Program; ARP-9949; contributed by Li Wu and Vineet N. KewalRamani, HIV Drug Resistance Program, National Cancer Institute, Frederick, Maryland, USA) were cultured in RPMI (Gibco;11875-119) supplemented with 10% FBS and 1% pen/strep (R10). THP-1 cells were differentiated with 100 nM PMA (Sigma-Aldrich) for 48 hours as described previously (40). THP-1 cells were transduced with lentivectors (100 ng p24^gag^ per 0.5 × 10^6^ cells) expressing shRNAs (Table 1). Transduced cells were maintained and selected in the presence of 2 μg/mL puromycin (InvivoGen). Validating knockdown cell lines at the functional level were carried out by incubating PMA/THP-1 cells with RLR or TLR ligands for 24–48 hours and measuring IP-10 in the culture supernatants via ELISA. Ligands used included 3p-hpRNA (2.5 ng/mL; InvivoGen; tlrl-hprna), LPS (TLR4 ligand; 100 ng/mL; InvivoGen; tlrl-eblps), or high molecular weight poly I:C (TLR3 ligand; 10 μg/mL; InvivoGen; tlrl-pic). Human MDMs were derived from CD14^+^ monocytes isolated from PBMCs by anti-CD14 antibody–conjugated magnetic beads (Miltenyi Biotech) and cultured in RPMI supplemented with 10% Human AB Serum (Sigma-Aldrich) and 20 ng/mL M-CSF (Peprotech; 300-25) for 6 days as previously described (29, 40).

### Viruses.

VSV-G–pseudotyped, single-round HIV-1 was generated by transient transfection of HEK293T cells as previously described (29). Virus titer was measured in TZM-bl cells (103). Lentivectors expressing shRNA were generated by cotransfecting HEK293T cells with pLKO.1, psPAX2, and H-CMV-G using calcium phosphate (102). Virus-containing supernatants were harvested 2 days after transfection, passed through 0.45 μm filters, and concentrated on a 20% sucrose cushion (100,000*g*, 4°C, 2 hours) with a SW28 rotor (Beckman Coulter). Virus pellets were resuspended in 1× PBS, aliquoted, and stored at –80°C until use. Lentiviral p24^gag^ content was measured via p24^gag^ ELISA as previously described (102). HSV-1 and Sendai viruses were provided by Mohsan Saeed (Boston University Chobanian & Avedisian School of Medicine, Boston, Massachusetts).

### Infections.

THP-1/PMA macrophages were infected with single-cycle HIV-1 reporter viruses (MOI 2) in the presence of polybrene (10 μg/mL; Millipore Sigma; TR-1003-G) via spinoculation (1,000*g*, room temperature, 1 hour). In some experiments, THP-1/PMA macrophages were pretreated (20 minutes) with EFV (1 μM; NIH AIDS Reference Reagent Program), raltegravir (30 μM; Selleck Chemical; 50-615-1), spironolactone (100 nM; Selleck Chemical; S4054), and KPT 330 (1 μM; Selleck Chemical; 50-136-5156). MDMs were pretreated with 2.5 mM dNs (Sigma-Aldrich) (104–106) for 2 hours and then infected with LaiΔenvGFP/G, LaiΔenvGFP-M10/G (MOI 1), or Lai-YU2env (MOI 1) as previously described (29) in the presence or absence of HIV-1 inhibitors.

### siRNA transfection of primary MDMs.

MDMs (1.5 × 10^6^ cells/well in a 6-well plate) were transfected with SMARTPool siRNA (25–50 nM; Horizon) (Table 2) via Trans-IT X2 (Mirius Bio; MIR6004) in Opti-MEM (Gibco). Transfected cells were detached with enzyme-free cell dissociation buffer (Millipore; S014B) at 24 hours after transfection and reseeded for infections as described above.

### RNA isolation and RT-qPCR.

Total RNAs were extracted using the RNeasy kit (Qiagen). cDNA was generated using Superscript III First-Strand cDNA Synthesis kit (Invitrogen), and qPCR was done using Maxima SYBR Green (Thermo Fisher Scientific). The C_T_ value of target mRNA was normalized to that of GAPDH mRNA (ΔC_T_), and ΔC_T_ of the target mRNA was further normalized to that of a control sample by the 2^–ΔΔC^_T_ method as described (107, 108). Primer sequences for GAPDH, IP-10, IFN-α, and IFN-β have been described previously (29). Primer sequences to assess expression of specific targets are listed in Table 3.

### Flow cytometry.

THP-1/PMA macrophages were detached using enzyme-free dissociation buffer (Millipore; S014B), while MDMs were detached using CellStripper (Corning; MT-25-056CI), washed once with 1× PBS, and fixed in 4% PFA for at least 30 minutes. Cells were analyzed on LSRII flow cytometer (BD Biosciences). Data were analyzed using FlowJo software.

### Western blot.

Cell lysates (30 μg) were analyzed by Western blotting using the following antibodies: rabbit anti-MAVS (Invitrogen; PA5-17256; 1:1,000), rabbit anti-IRF3 (Cell Signaling; 4302S; 1:1,000), rabbit anti-IRF5 (Cell Signaling; 76983S; 1:1,000), rabbit anti-IRF7 (Cell Signaling; 4920S; 1:1,000), rabbit anti-TRAF6 (Cell Signaling; 8028,;1:1,000), rabbit anti-IKK-β (Cell Signaling; 2684S; 1:1,000), mouse anti-actin antibody (Thermo Fisher Scientific; AM4302; 1:5,000), goat anti-mouse IgG secondary antibody Dylight 680 (Thermo Fisher Scientific; SA5-35518; 1:10,000), and goat anti-rabbit IgG secondary antibody Dylight 800 (Thermo Fisher Scientific; SA5-35571; 1:10,000). Membranes were scanned using an Odyssey scanner (Li-Cor).

### Immunofluorescence and microscopy.

THP-1/PMA macrophages were cultured and infected on coverslips (Fisher Scientific;12-541-001) in 12-well plates. At 3 dpi, cells were fixed using 4% PFA (Boston Bioproducts; BM-155) for 30 minutes at 4°C, washed once with PBS, and permeabilized using 0.1% Triton X-100 (Cayman Chemical; NC1636886) in PBS for 5 minutes at room temperature. Cells were stained with anti-IRF5 antibody (Cell Signaling; 76983S) and Alexa594-conjugated goat anti-mouse antibody (Invitrogen) and counterstained with DAPI (Sigma-Aldrich; D1306). Cells were mounted on slides using Fluoromount medium (Southern Biotech; OB100-01). Images were acquired with an EVOS M5000 microscope (Invitrogen; A40486) or a Leica SP5 confocal microscope and analyzed using ImageJ (NIH) or CellProfiler (109).

### RNA coimmunoprecipitation assay.

HEK293T cells were infected with LaiΔenvGFP/G at MOI 1 and transfected 18 hours after infection with 3 μg of MDA5-Flag plasmid or 0.1 μg of RIG-I–Flag plasmid (+filler plasmid). The next day, cells were washed twice with PBS and UV cross-linked at 40 mJ/cm^2^. Cells were lysed with 400 μL of fractionation buffer (Life Technologies; 4403461) supplemented with protease inhibitor (Roche; 11836170001), RNAseOUT (100 U/mL; Invitrogen; 10777019), and DTT (5 μL/mL; Invitrogen; 18080051). Forty microliters of lysate was saved as input, and the remaining cytoplasmic lysate was incubated with antibody-coupled beads as described previously (110). Beads (Invitrogen; 61-011-LS) were coated with 2 μg of anti-Flag antibody (Sigma; F3165) or IgG control (Novus Biologicals; NBP1-97019). Following overnight antibody coupling, beads were washed using high-salt buffers as described previously (111). RNA extraction, cDNA synthesis, and qPCR were carried out as previously described (110).

### ELISA.

IP-10 in supernatants was measured with ELISA according to the manufacturer’s protocol (BD Biosciences; 550926).

### Nanostring analysis.

Total RNA was isolated from infected MDMs using an RNeasy kit (Qiagen; 74106) and quantified via a NanoDrop spectrophotometer (Thermo Scientific). Samples were analyzed using the Human Myeloid Innate Immunity V2 Panel (Nanostring; 115000171) on the nCounter system. Nanostring counts were analyzed using nSolver; raw counts of all targets were normalized to the geometric mean of positive controls, and housekeeping controls included in the Nanostring Myeloid Innate Immunity Panel. Counts between donors were compared using 2-tailed *t* test, and data were plotted as log_2_(fold change) versus –log(*P* value) with the lines representing a *P* value of 0.05.

### Study population.

PBMCs were derived from the HIV/Aging Cohort established at BMC (15). The cohort recruited people without HIV in 2 age-stratified groups: younger (18–35 yrs) and older (≥50 yrs). Donors with active hepatitis B or C or recent immunomodulatory therapy (oral or injected corticosteroids, plaquenil, azathioprine, methotrexate, biologic therapies, systemic or local IFN, chemotherapy, or HIV vaccine) were excluded (15). PBMCs were also isolated from leukopaks (NY Biologics) from anonymous donors stratified by age into younger (18–35 yrs) or older (≥50 yrs).

### Statistics.

Statistical analysis was performed using GraphPad Prism 10. *P* values were calculated via 1-way ANOVA with Tukey’s or Dunnett’s post test for multiple-comparison analysis or by unpaired 2-tailed *t* test.

### Study approval.

All studies were approved by the Boston University Medical Center Institutional Review Board, Boston, Massachusetts, USA.

### Data availability.

Raw data are available in the Supporting Data Values file.

## Author contributions

SR, HA, and SG designed the experiments. SR, AAQM, and JB performed the experiments and analyzed the data. AJO, YC, YL, AA, and MS assisted in obtaining and isolating PBMCs from participants of the HIV/Aging Cohort (BMC). AJH and MS edited the manuscript. SR, HA, and SG wrote the manuscript.

## Supplementary Material

Supplemental data

Unedited blot and gel images

Supporting data values

## Figures and Tables

**Table 3:**
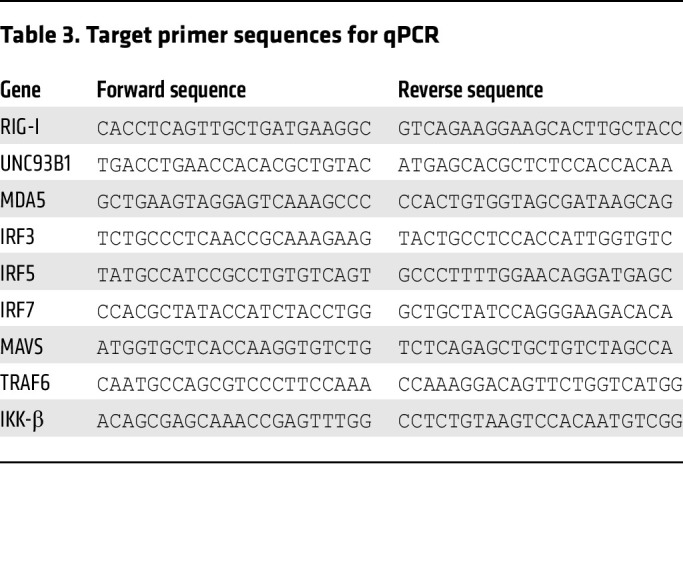
Target primer sequences for qPCR

**Table 2:**
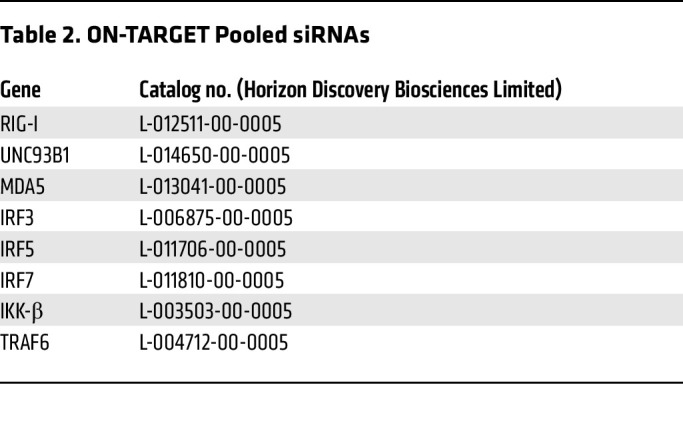
ON-TARGET Pooled siRNAs

**Table 1:**
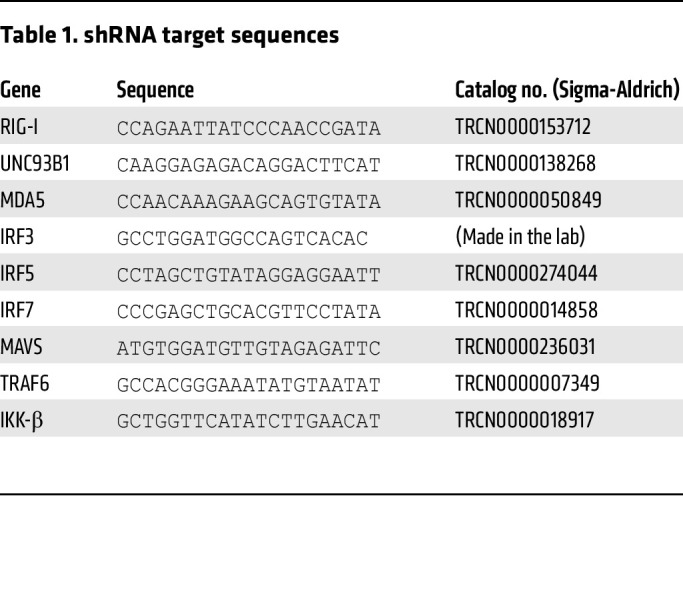
shRNA target sequences

## Version 1 06/10/2025

In-Press Preview

## Version 2 08/15/2025

Electronic publication
