# Supplementary material for: The macrophage-intrinsic MDA5/IRF5 axis drives HIV-1 intron-containing RNA-induced inflammatory responses
Source: J Clin Invest. 2025 Jun 10;135(16):e187663. doi: 10.1172/JCI187663 (PMC12352897; doi:10.1172/JCI187663)
Supplement: Supplemental data [file jci-135-187663-s212.pdf]

Supplemental Material

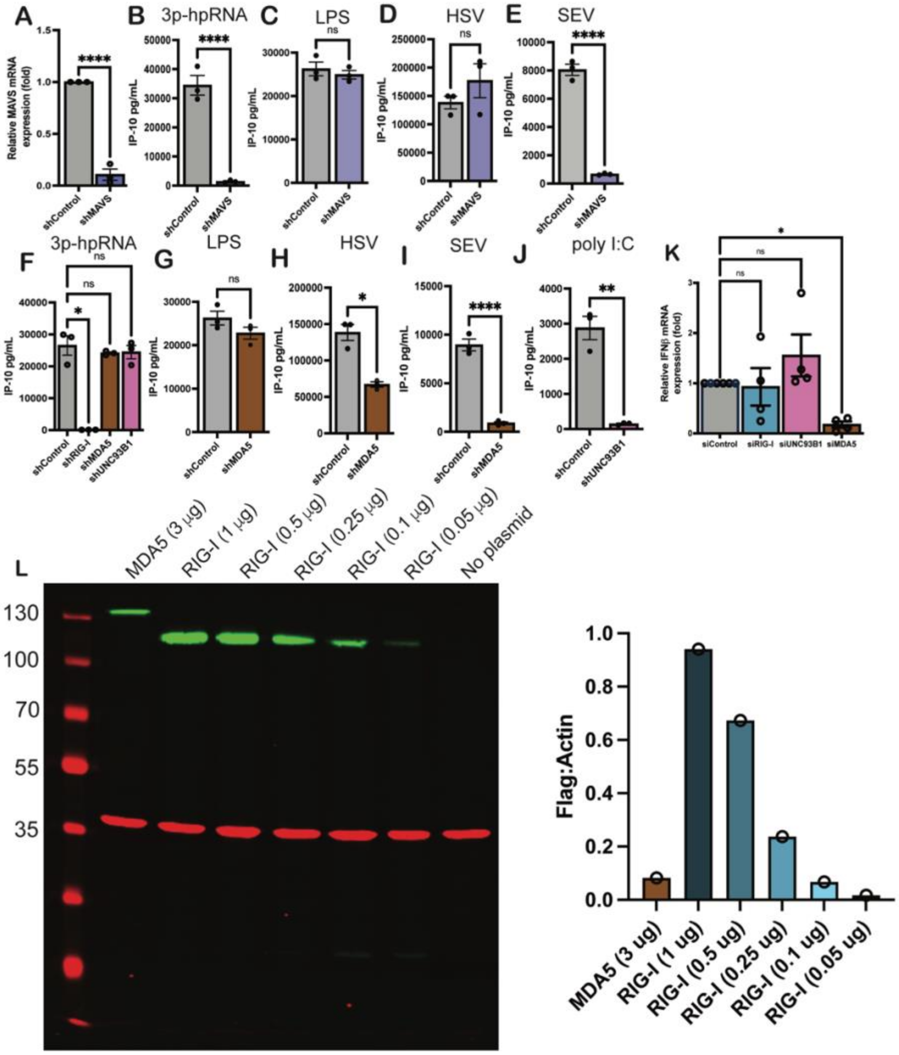

<u>**Supplementary Figure 1**</u> **(A)** Knockdown of MAVS expression in THP-1/PMA macrophages was validated using RT-qPCR. **(B-E)** MAVS kd THP-1/PMA cells were treated with 3p-hpRNA(2.5 ng/mL), LPS(100 ng/ml), infected with HSV (MOI 0.1) or Sendai virus (SEV)(MOI 1), and supernatants were harvested 24 h post-stimulation or 48 hpi for analysis of IP-10 production via ELISA. **(F-J)**THP-1/PMA cells with knock-down of RIG-I/MDA5/UNC93B1 expression were treated with **(F)**3p-hpRNA(2.5 ng/mL), **(G)**LPS(100 ng/mL), or infected with **(I)**HSV (MOI 0.1) or **(I)**Sendai virus (MOI 1), or treated with **(J)**HMW polyI:C(10 mg/ml). Supernatants were harvested 24 hours post-stimulation or 48 hpi for analysis of IP-10 production via ELISA. **(K)**siRNA-transfected MDMs were infected with Lai∆env GFP/G (MOI 1) in the presence of dNs and harvested for analysis of IFNβ mRNA expression via RT-qPCR at 2 dpi. **(L)**HEK293T cells were transfected with varying concentrations of RIG-I-Flag, or a single amount of MDA5-Flag expression plasmids. Expression of MDA5/RIG-I-Flag was assessed via Western blot . Flag intensity was normalized to that of actin and plotted as a graph. Data is displayed as mean ± SEM with each dot representing an experiment **(A-J)** or individual donor **(K)**. Statistical significance assessed via unpaired t-test **(A-E,F-J)** or 1-way ANOVA **(K)** with Dunnett's multiple comparisons analysis. *: $p < 0.05$; **: $p<0.01$, ****: $p < 0.0001$,ns = not significant.

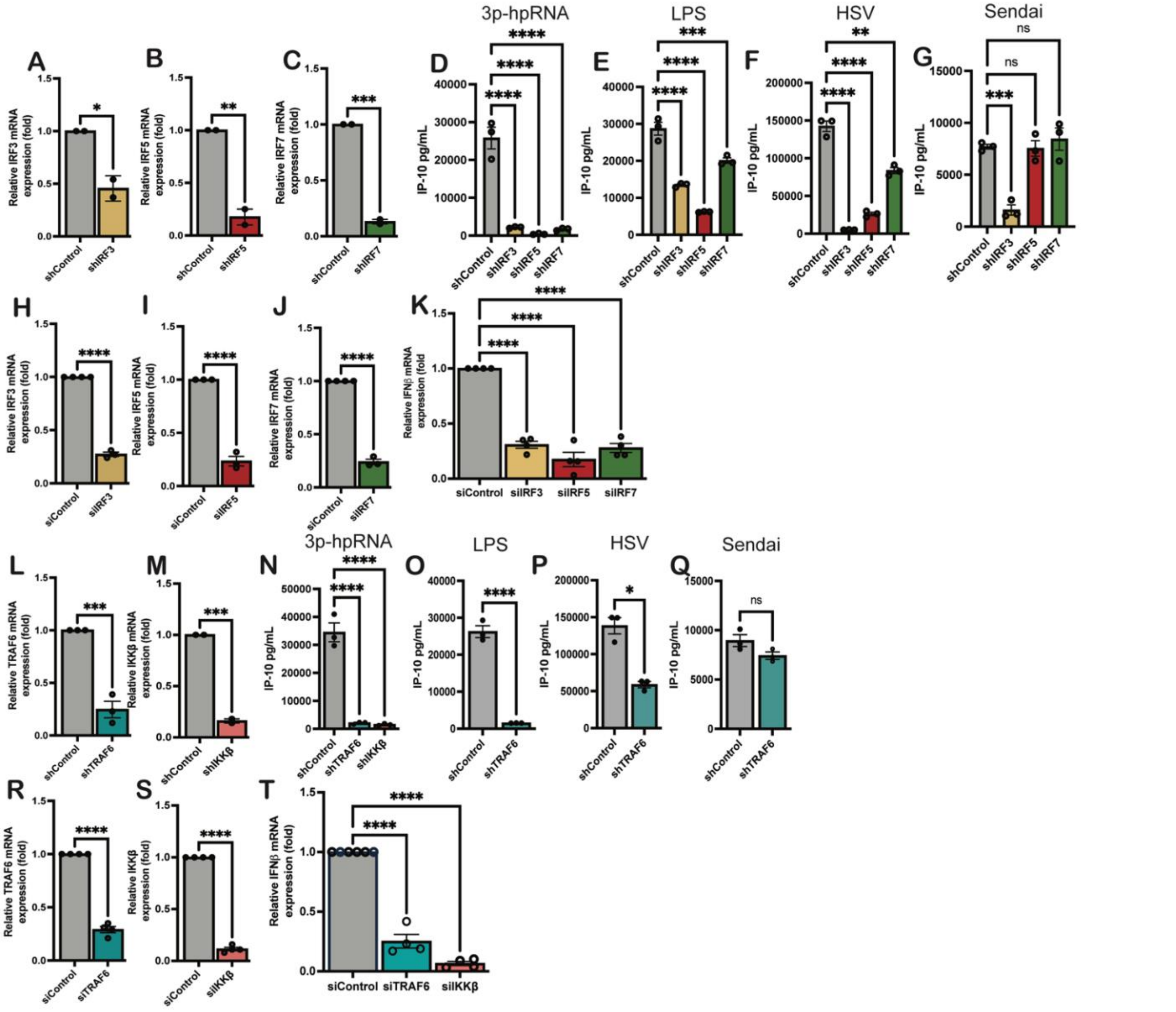

<u>Supplementary Figure 2</u> Knockdown of **(A)**IRF3, **(B)**IRF5, or **(C)**IRF7 expression in THP-1/PMA macrophages was validated by RT-qPCR for (n=3). Functional knockdown of IRF expression in THP1/PMA macrophages was validated using **(D)**3p-hpRNA(2.5 ng/ml), **(E)**LPS(100ng/ml), **(F)**HSV (MOI 0.1) infection or **(G)**Sendai virus (MOI 1) infection. MDMs were transfected with siRNA targeting **(H)**IRF3, **(I)**IRF5, or **(J)**IRF7 for 48 hours and knockdown validated using RT-qPCR(n=4). siRNA transfected MDMs were infected with LaiΔenvGFP/G (MOI 1) in the presence of dNs and harvested for analysis of **(K)**IFNβ mRNA expression via RT-qPCR at 2 dpi. Knockdown of **(L)**TRAF6 and **(M)**IKKβ expression in THP-1/PMA macrophages was validated using RT-qPCR, and functionally validated by treatment with **(N)**3p-hpRNA(2.5 ng/mL), **(O)**LPS(100 ng/mL), infection with **(P)**HSV (MOI 0.1) or **(Q)**Sendai virus (MOI 1). Cell supernatants were harvested 24 h post-stimulation or 2 dpi for analysis of IP-10 production via ELISA. **(R-S)**MDMs were transfected with siRNA against **(R)**TRAF6 or **(S)**IKKβ and mRNA expression was measured by RT-qPCR (n=4).**(T)**MDMs transfected with siRNA targeting TRAF6 or IKKβ and infected with LaiΔenvGFP/G (MOI 1) in the presence of dNs and harvested for analysis of IFNβ mRNA expression at 2 dpi. Data is represented as mean ± SEM with each dot representing an individual experiment**(A-G,L-Q)** or donor**(K,T).** Statistical significance was assessed using unpaired t-test **(A-C,H-J,L-M,O-S)** or 1-way ANOVA with Dunnett's multiple comparisons analysis **(D-H,K,N,T).** *: $p < 0.05$; **: $p<0.01$, ***: $p < 0.001$ ****: $p < 0.0001$, ns = not significant.

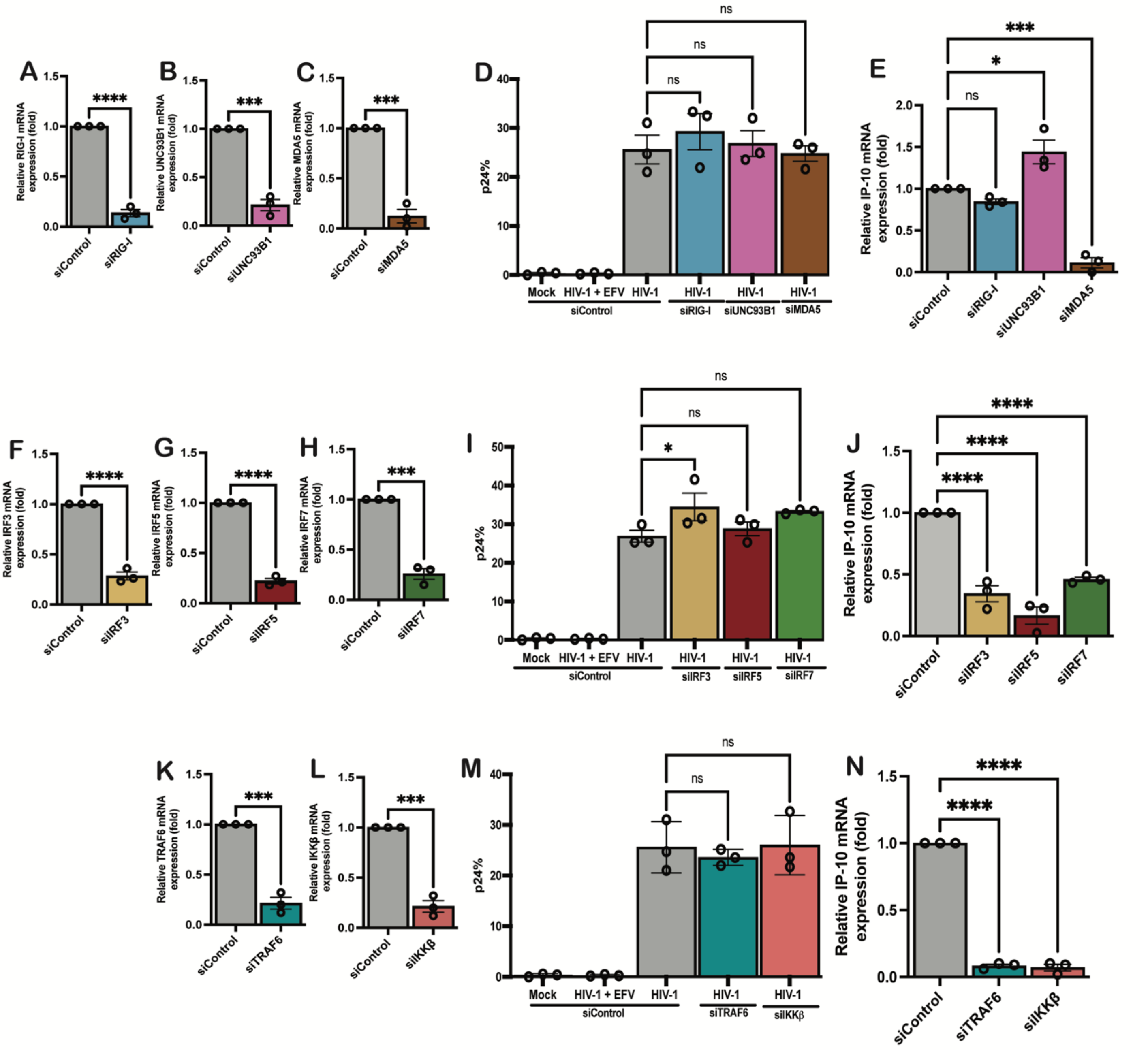

__Supplementary Figure 3__ MDMs were transfected with siRNA targeting **(A)**RIG-I, **(B)**UNC93B1, or **(C)**MDA5 for 48 hours and knockdown validated using RT-qPCR (n=3). siRNA transfected MDMs were infected with replication competent Lai-YU2env (MOI 1) in the presence of dNs and harvested at 2 dpi. Infection was measured by intracellular p24 staining via **(D)**flow cytometry and **(E)**IP-10 expression was measured by RT-qpCR. MDMs were transfected with siRNA targeting **(F)**IRF3, **(G)**IRF5, or **(H)**IRF7 for 48 hours and knockdown validated using RT-qPCR (n=3). siRNA transfected MDMs were infected with replication competent Lai-YU2 (MOI 1) in the presence of dNs and harvested at 2 dpi for assessment of **(I)**infection and **(J)**IP-10 expression. MDMs were transfected with siRNA targeting **(K)**TRAF6 or **(B)**IKKβ for 48 hours and knockdown validated using RT-qPCR(n=3). siRNA transfected MDMs were infected with replication competent Lai-YU2 (MOI 1) in the presence of dNs and harvested at 2 dp for assessment of **(M)**infection and **(N)** IP-10 expression. Data is represented as mean ± SEM with each dot representing an individual donor (A-N). Statistical significance was assessed using unpaired t-test **(A-C,F-J,K-L)** or 1-way ANOVA with Dunnett's multiple comparisons analysis **(D-E,I-J,M-N).** *: $p < 0.05$, ***: $p < 0.001$ ****: $p < 0.0001$,ns = not significant.

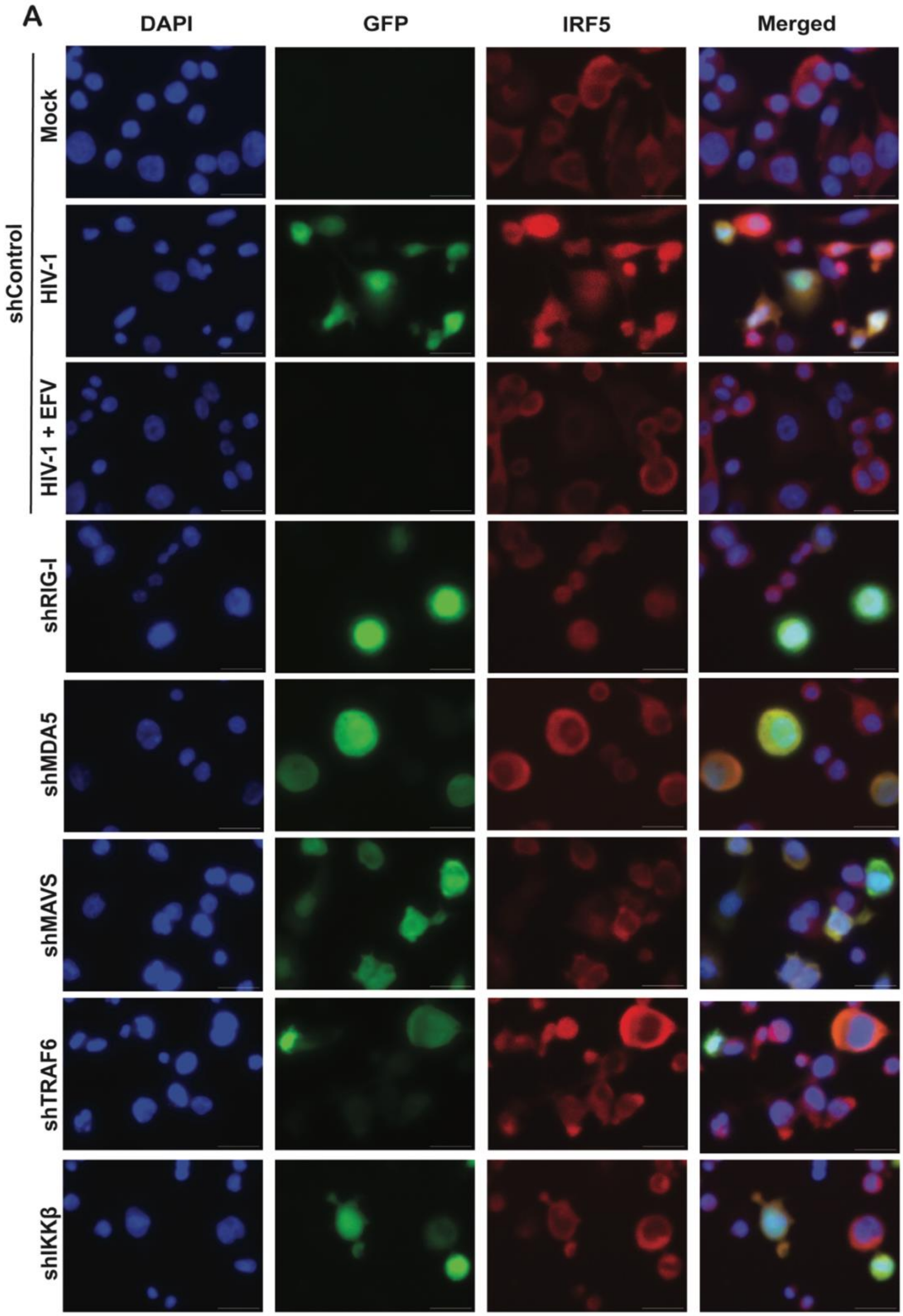

Supplementary Figure 4 THP1/PMA macrophages transduced with lentivectors expressing shRNAs were infected with LaiΔenvGFP/G (MOI 2) ± EFV (1μM) on coverslips. Cells were fixed at 3 dpi and stained to visualize intracellular IRF5 localization and DAPI via immunofluorescence imaging. Scale bar = 100 μm.

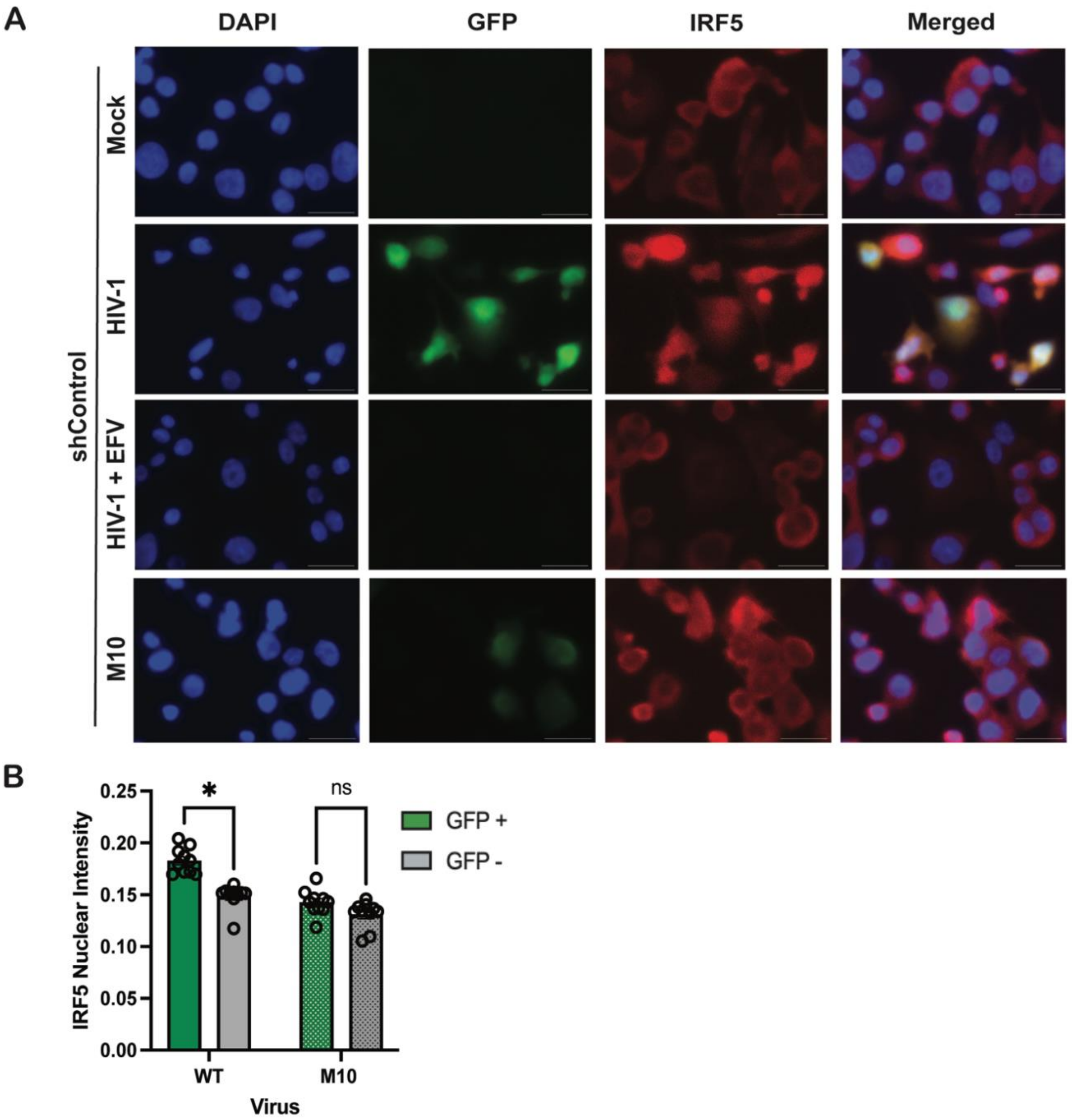

Supplementary Figure 5 **(A)**THP-1/PMA macrophages were infected with LaiΔenvGFP/G (WT or M10 mutant) viruses (MOI 2). At 3 dpi, cells were fixed and stained for IRF5 localization and DAPI to visualize nuclei. Scale bar = 100 μm. **(B)**Cell Profiler was utilized to assess IRF5 nuclear intensity in infected and uninfected cells Images from three independent infection experiments were quantified, with each dot representing a field containing approximately 50-150 cells. Representative images for shControl cells infected with WT virus are the same as those in Figure S4. Statistical significance assessed via unpaired t-test**(B)**. *: p < 0.05,ns = not significant.

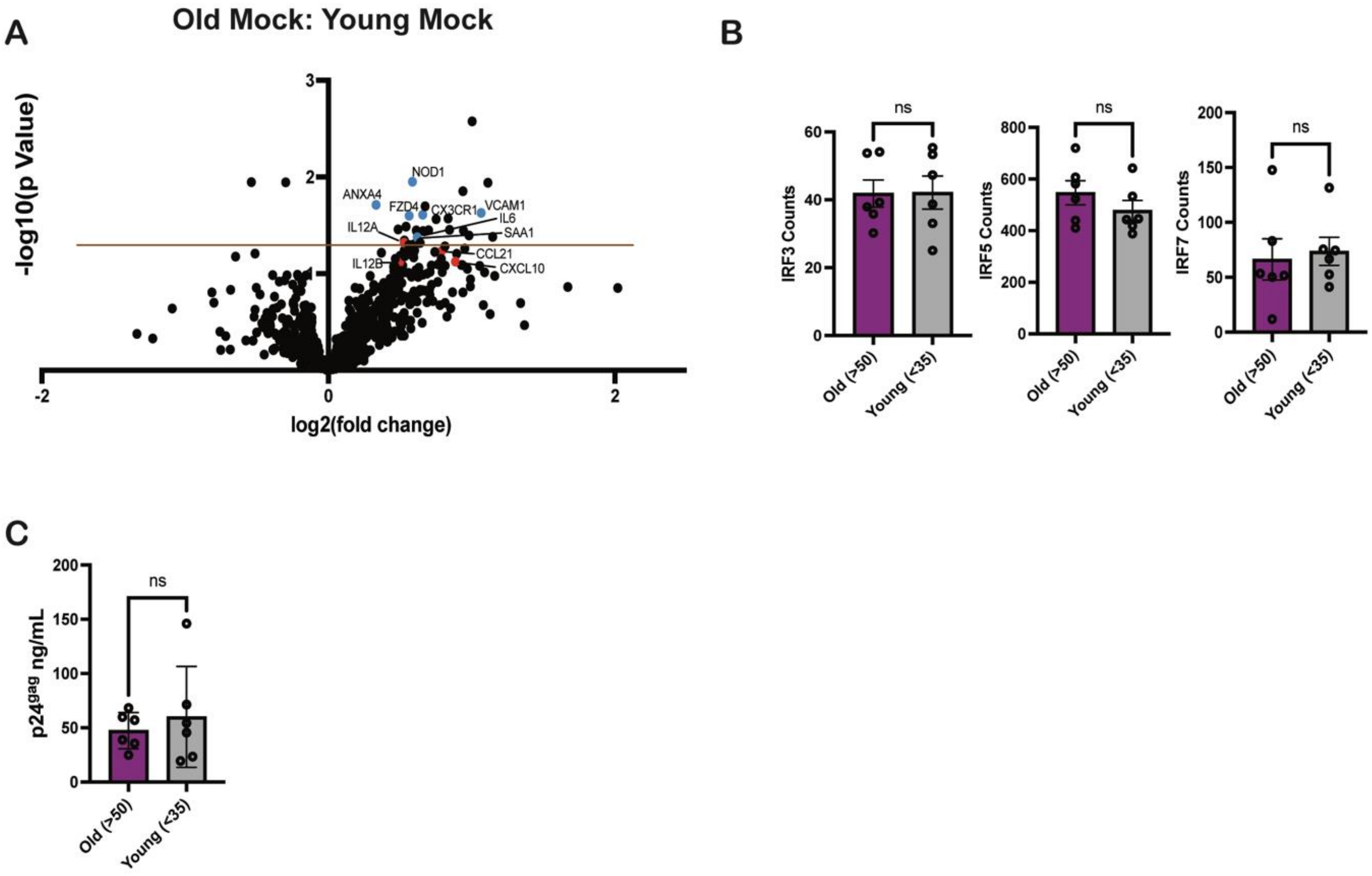

<u>Supplementary Figure 6 (</u>A)RNA isolated from Lai∆envGFP/G-infected MDMs (MOI 2) was analyzed via Nanostring nCounter using the Myeloid Innate Immunity V2 panel. Baseline expression of the gene panel was calculated using nSolver and plotted as a ratio of Old (Mock) vs. Young (Mock) with significantly upregulated IRFs (red) highlighted. The dashed line represents p-value of 0.05. **(B)**Raw count values for IRF3, IRF5, and IRF7 were plotted to assess differences in basal mRNA expression. **(C)** p24gag levels for selected donors in order to ensure equivalent levels of infection measured by ELISA. Significance was assessed via unpaired two-tailed t-test**(A-C)**. ns = not significant.

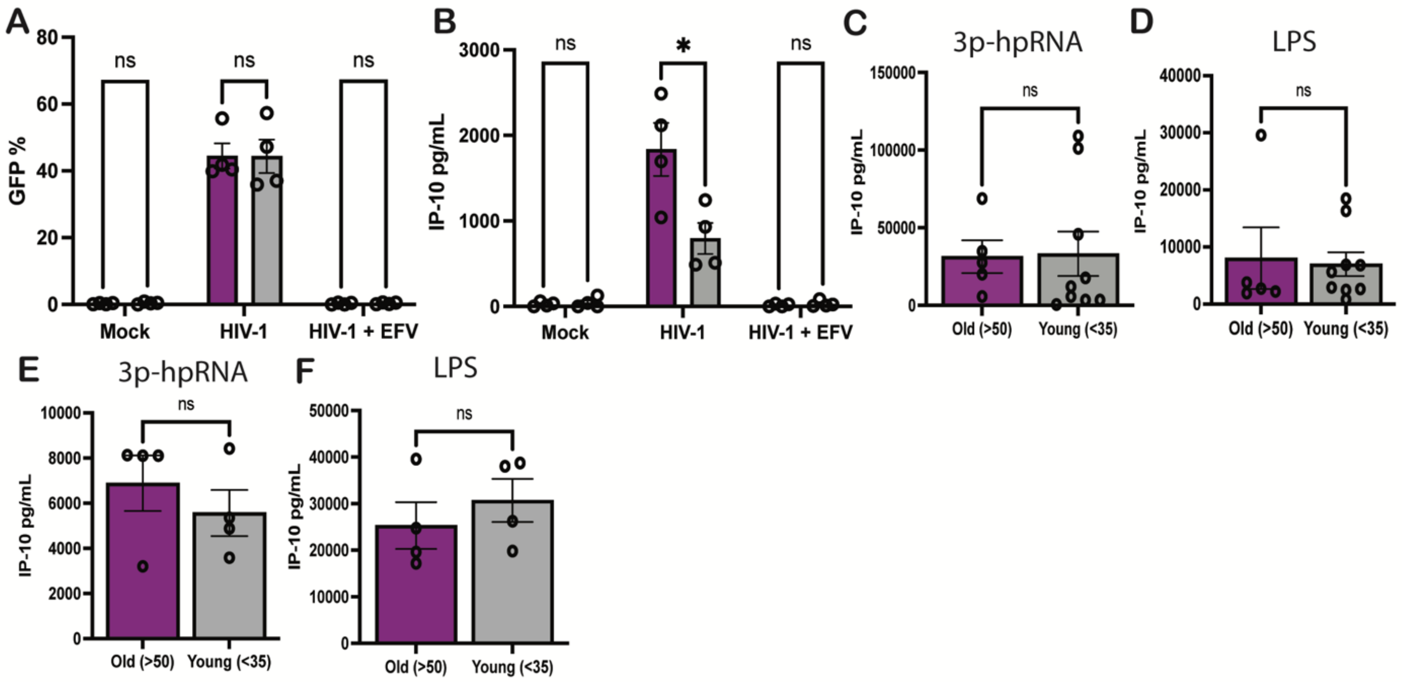

<u>Supplementary Figure 7</u> (A-B)MDMs (NY Biologics) were infected with Lai∆envGFP/G (MOI 1) in the presence of dNs and cells and supernatants were harvested at 3 dpi to assess **(A)**levels of infection and **(B)**IP-10 secretion. MDMs from **(C-D)**HIV/Aging cohortor **(E-F)**NY Biologics were treated with either 3p-hpRNA(2.5 ng/mL) or LPS(100 ng/mL) and supernatant was harvested at 18h post-stimulation to assess levels of IP-10 secretion by ELISA. Statistical significance was assessed via unpaired t-test**(A-F)** *: $p<0.05$,ns = not significant.

Supplementary Table 1

Group	Old (>50)	Young (<35)
Age (Mean ± SD)	64 ± 7.9	29.5 ± 4.2
Sex (% Male)	50	50
BMI (Mean ± SD)	28.5 ± 2.9	30.5 ± 7.9
Gastrointestinal Disorder (%)	66.7	16.7

Supplementary Table 2

Group	Old (>50)	Young (<35)
Age (Mean± SD)	71.75± 7.8	28.0 ± 4.8
Sex (% Male)	75	75

Table 1 summarizes characteristics of the HIV/Aging Cohort (BMC). Table 2 summarizes characteristics of leukopaks obtained from NY Biologics.
