# Supplementary material for: The macrophage-intrinsic MDA5/IRF5 axis drives HIV-1 intron-containing RNA-induced inflammatory responses
Source: J Clin Invest. 2025 Jun 10;135(16):e187663. doi: 10.1172/JCI187663 (PMC12352897; doi:10.1172/JCI187663)
Supplement: Unedited blot and gel images [file jci-135-187663-s213.pdf]

JCI Uncropped WBs

Unedited blot for Figure 1E

Lane 1: SCR THP-1/PMA
Lane 2: shMAVS THP-1/PMA

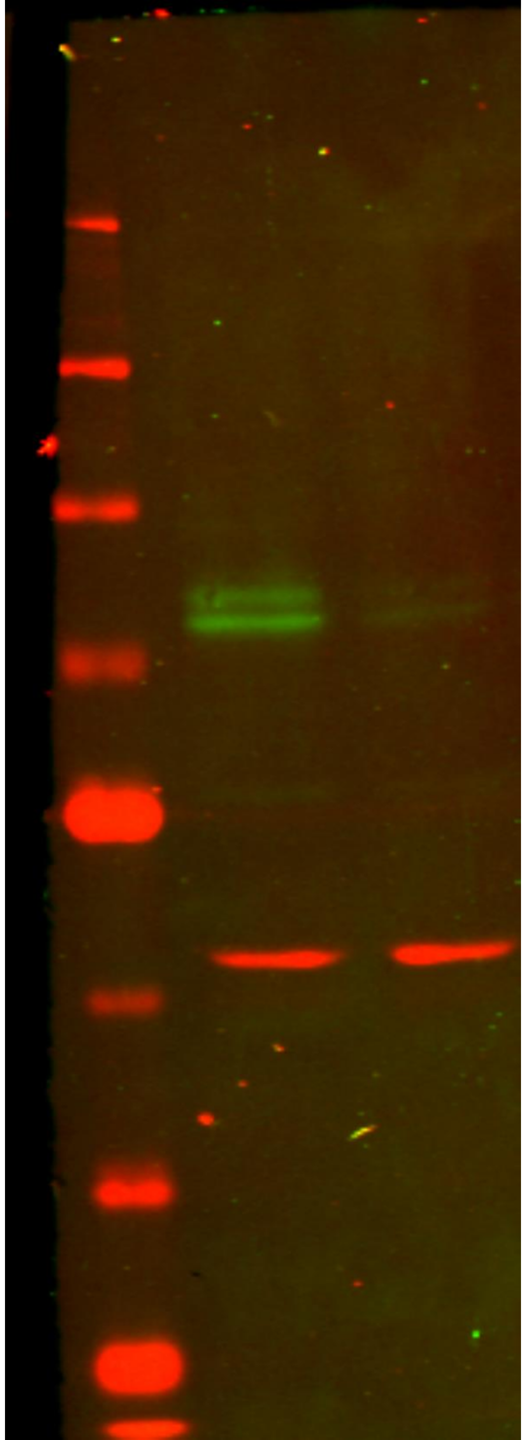

MAVS (75 kDa)

Actin (42 kDa)

Unedited blot for Figure 4A/D

Lane 1: Untransduced THP-1/PMA
Lane 2: shControl THP-1/PMA
Lane 3: shIRF3 THP-1/PMA
Lane 4: siControl MDM
Lane 5: siIRF3 MDM

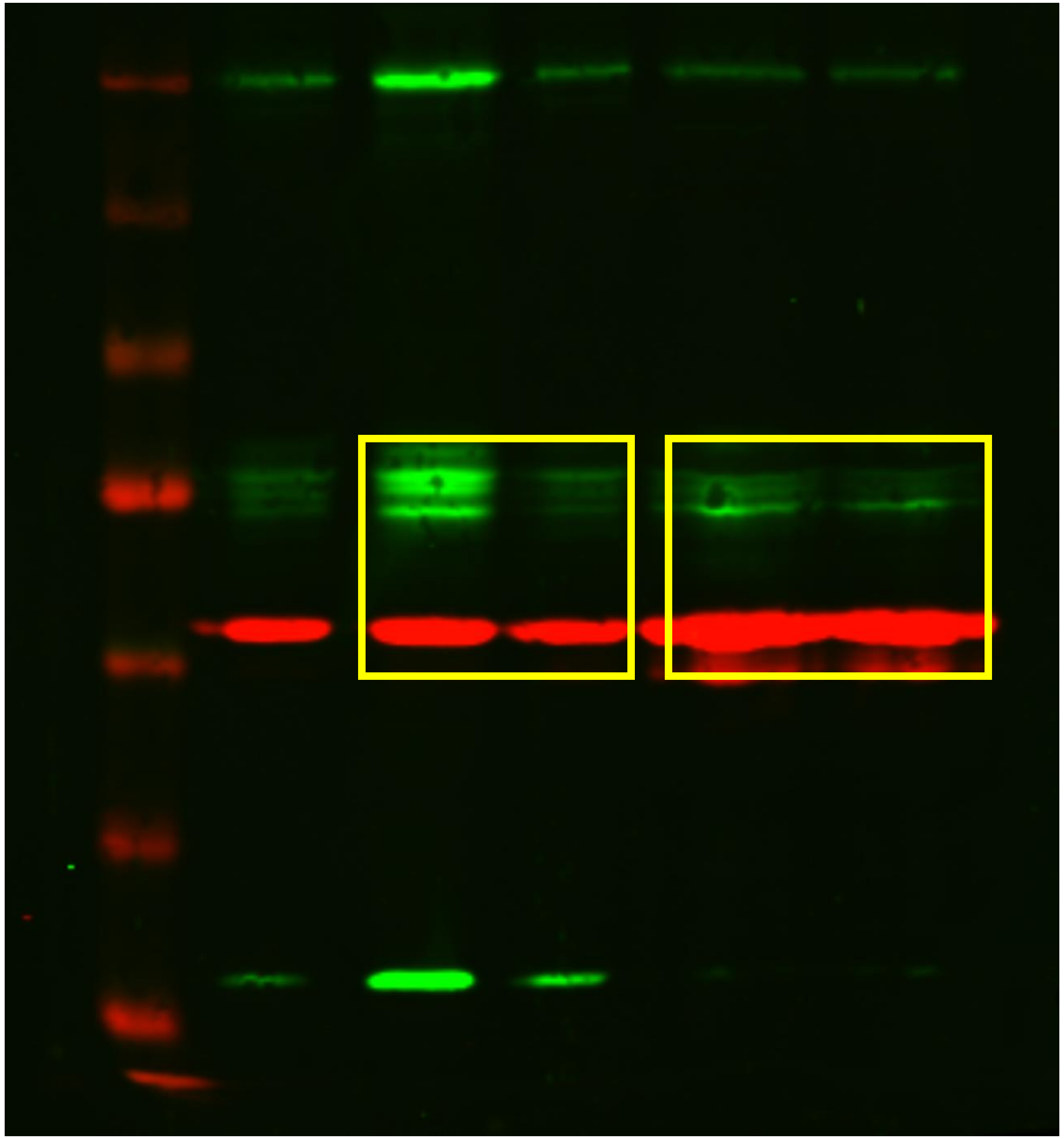

IRF3 (47-49 kDa)

Actin (42 kDa)

Unedited blot for Figure 4A/D

Lane 1: shControl THP-1/PMA
Lane 2: shIRF5 MDM THP-1/PMA

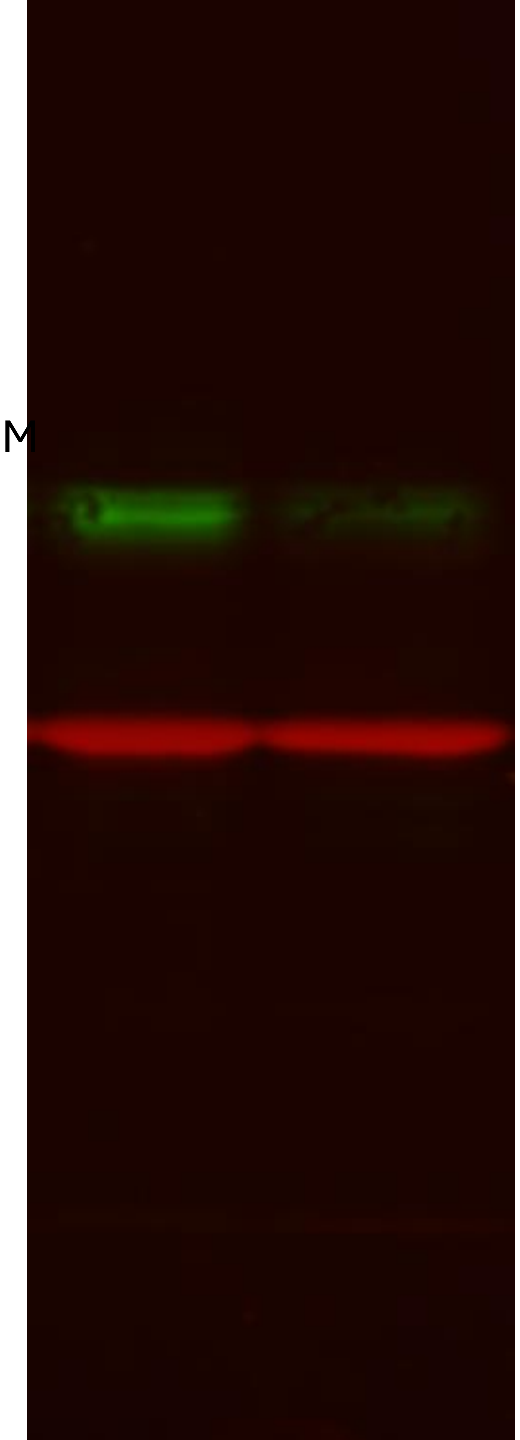

IRF5 (56 kDa)

Actin (42 kDa)

Lane 1: siControl MDM
Lane 2: siIRF5 MDM

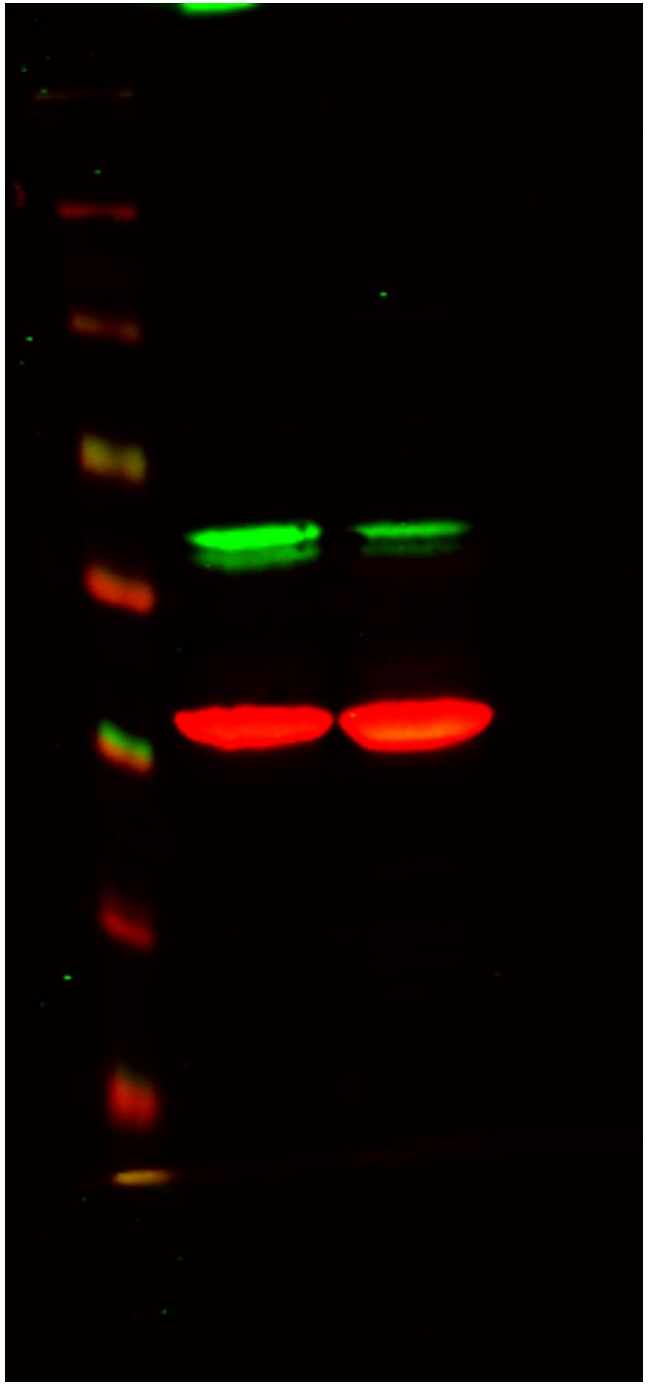

IRF5 (56 kDa)

Actin (42 kDa)

Unedited blot for Figure 4A/D

Lane 1: shScramble THP-1/PMA
Lane 2: shIRF7 THP-1/PMA

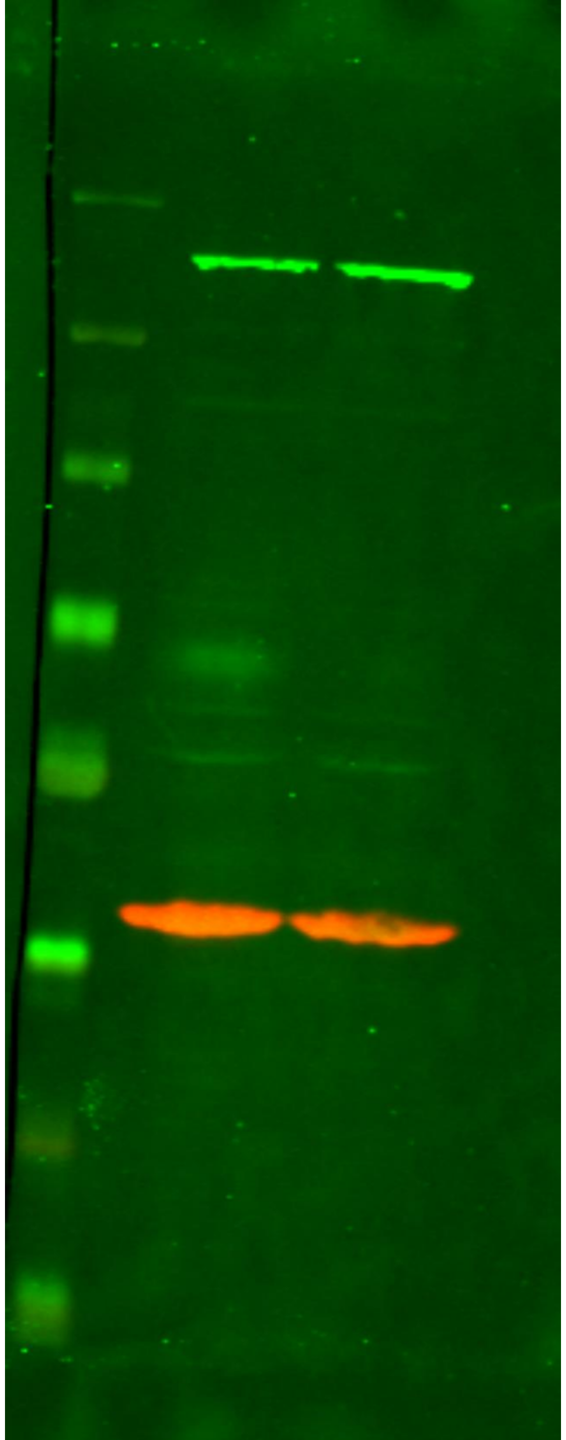

IRF7 (65 kDa)

Actin (42 kDa)

Lane 1: siControl MDM
Lane 2: siIRF7 MDM

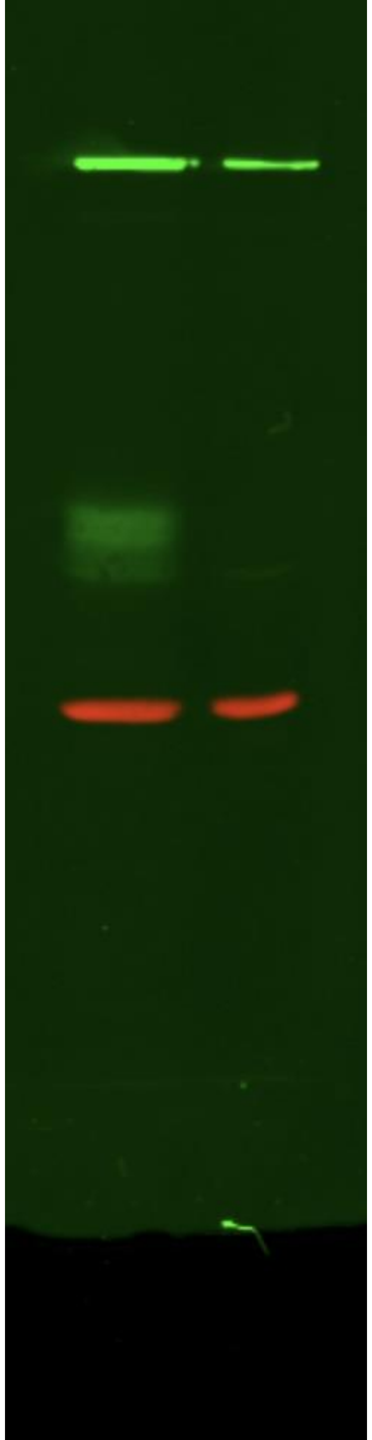

IRF7 (65 kDa)

Actin (42 kDa)

Unedited blot for Figure 5A/D

Lane 1: shControl THP-1/PMA
Lane 2: shTRAF6 THP-1/PMA
Lane 3: siControl MDM
Lane 4: siTRAF6 MDM

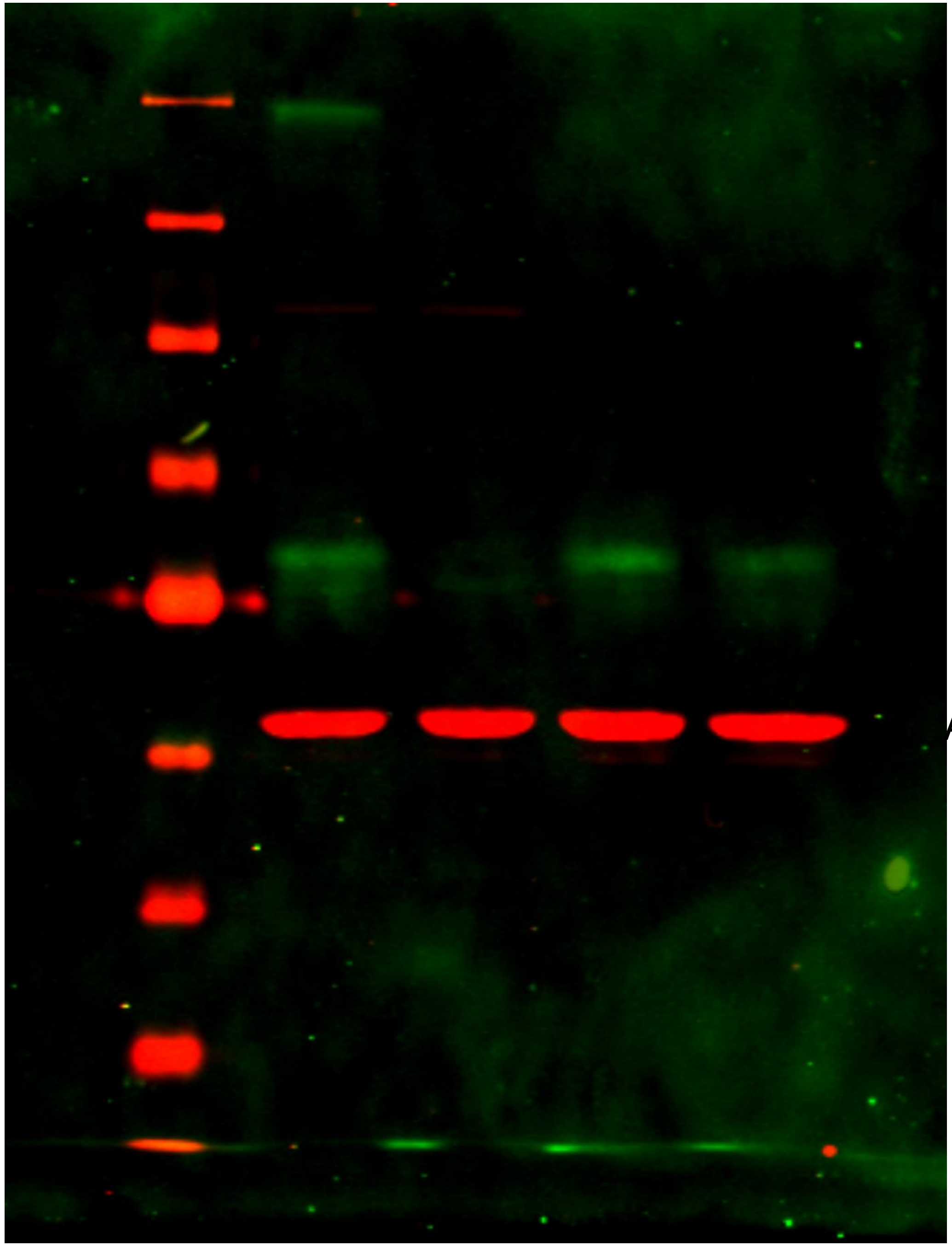

TRAF6 (60 kDa)

Actin (42 kDa)

Unedited blot for Figure 5A/D

Lane 1: shControl THP-1/PMA
Lane 2: sHIKKb THP-1/PMA

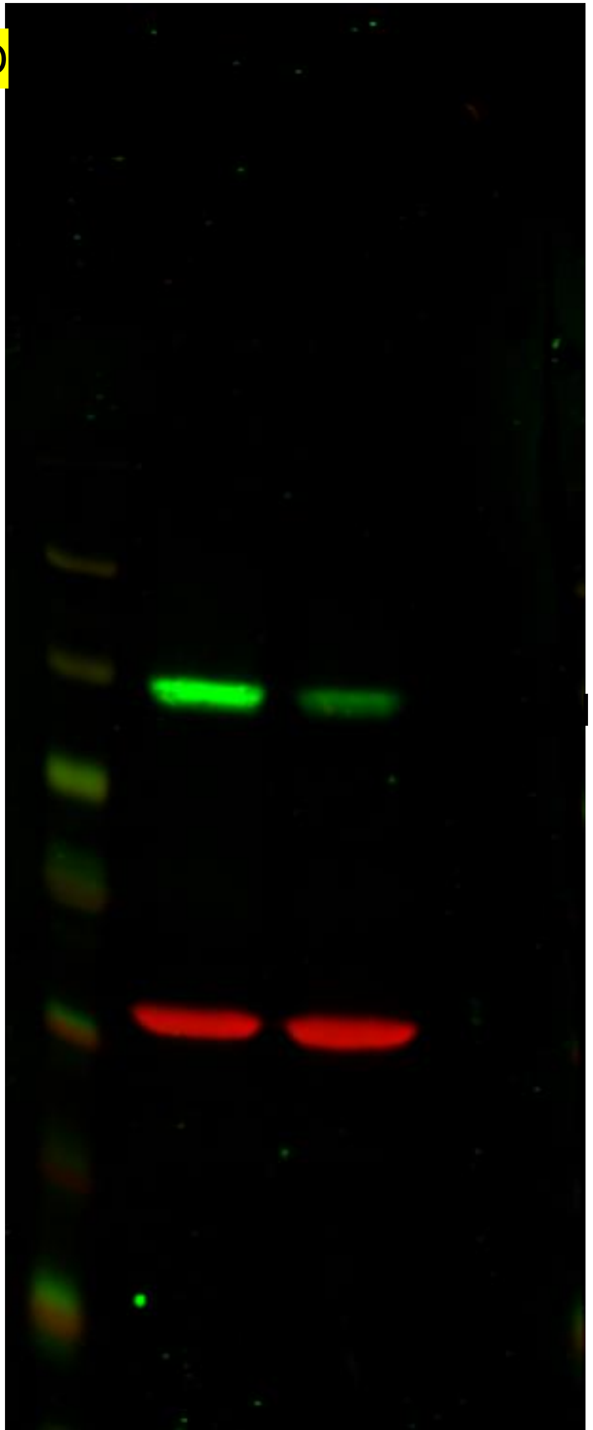

IKKb (87 kDa)

Actin (42 kDa)

Lane 1: siControl MDM
Lane 2: siIKKb MDM

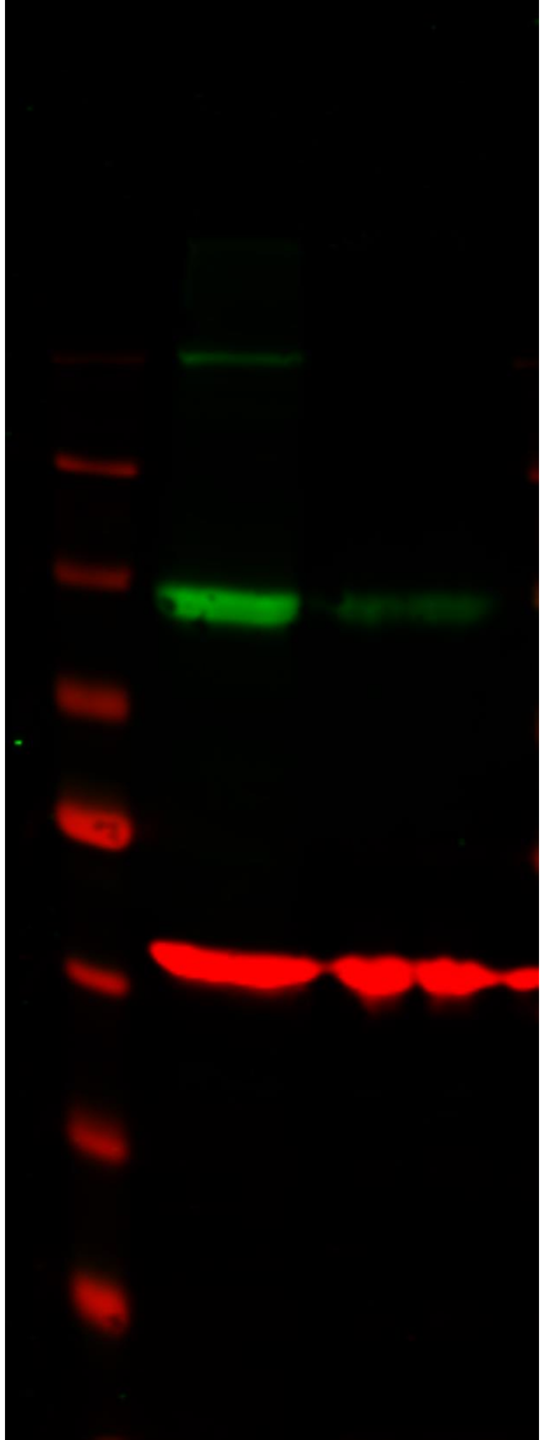

IKKb (87 kDa)

Actin (42 kDa)

Unedited blot for Figure 7G

Lane 1: THP/PMA (reference)
Lane 2: Old (>50) MDM
Lane 3: Young (<35) MDM

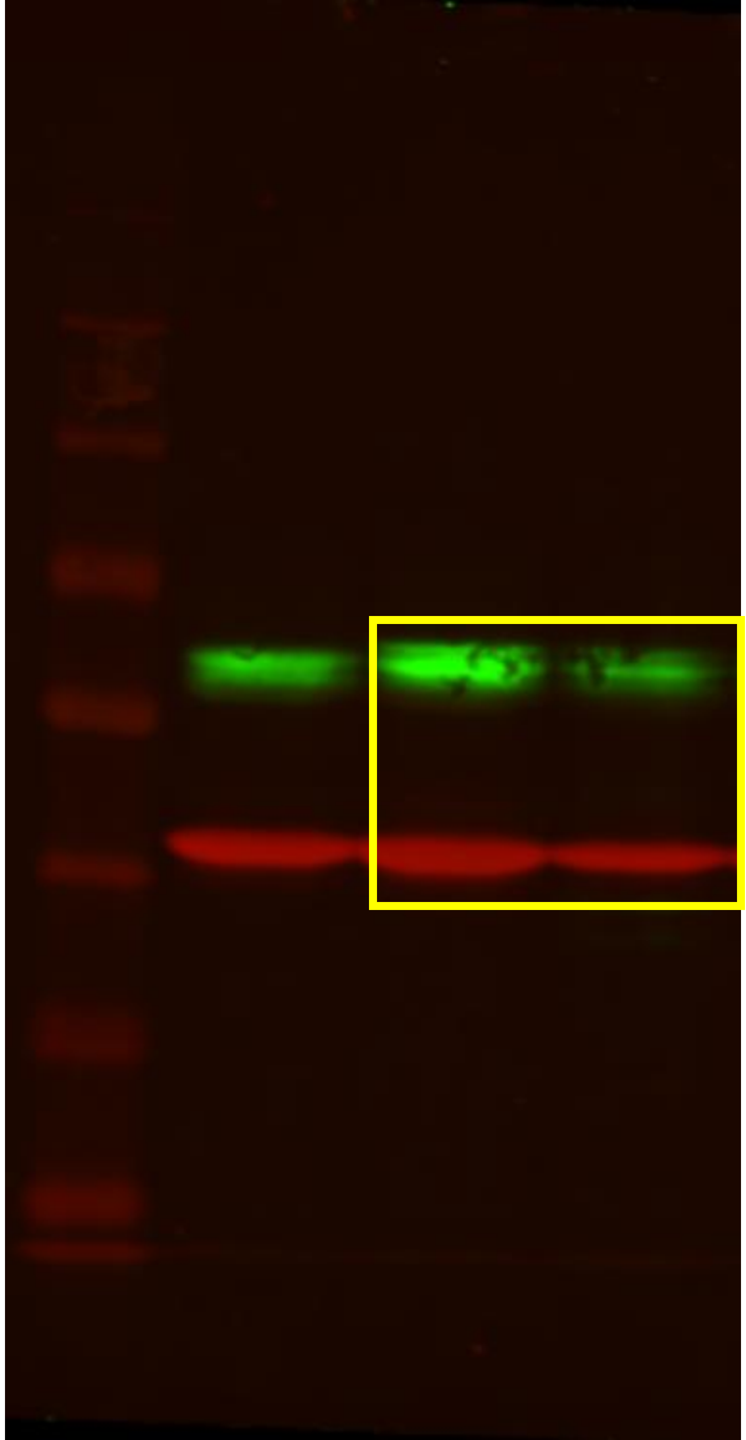

IRF5 (56 kDa)

Actin (42 kDa)

Unedited blot for Figure 7H

Lane 1: Old (>50) Monocyte
Lane 2: Young (<35) Monocyte
Lane 4: Old (>50) Monocyte
Lane 5: Young (<35) Monocyte

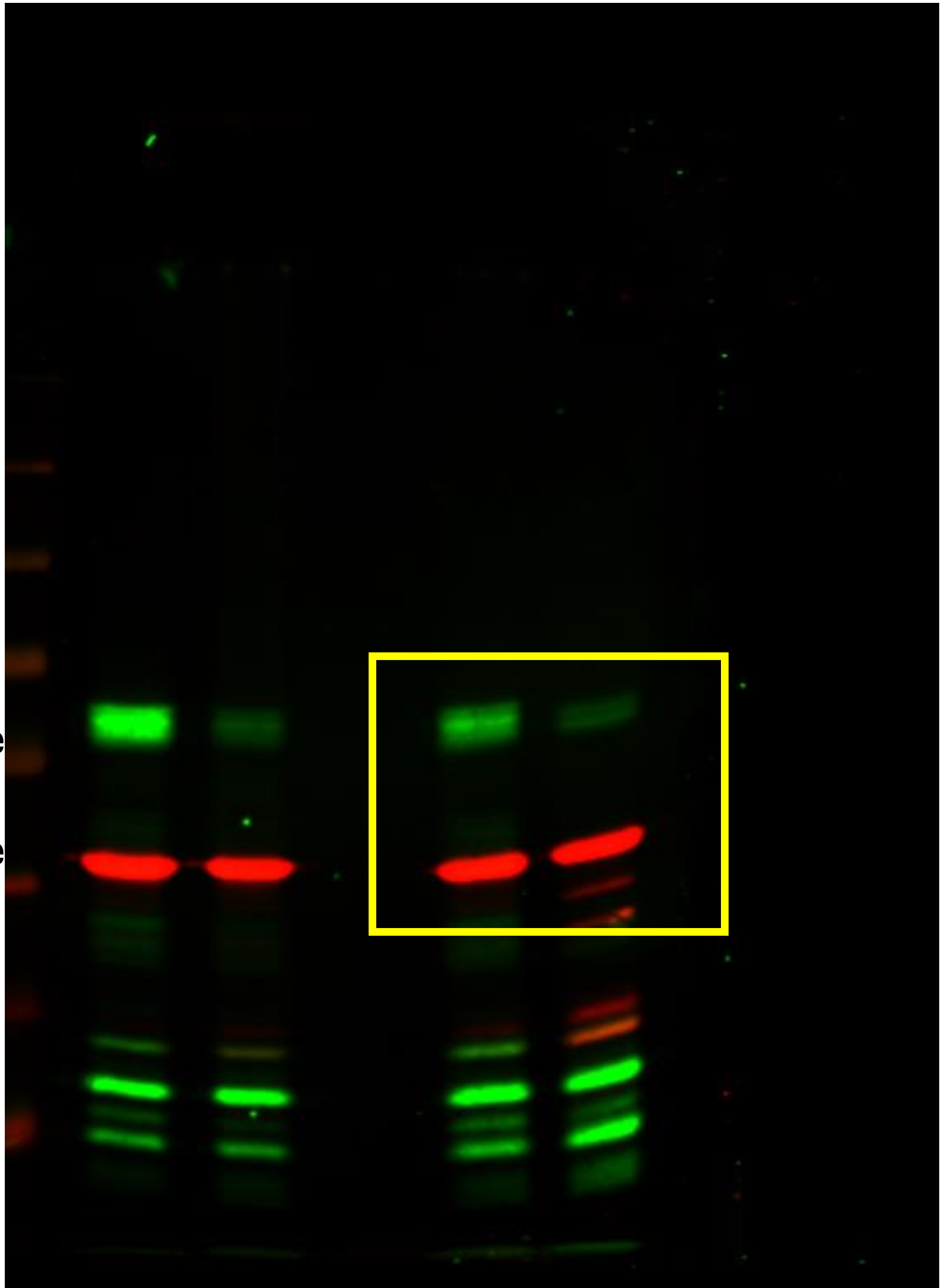

IRF5 (56 kDa)

Actin (42 kDa)
